# Heterostrain-enabled ultrahigh electrostrain in lead-free piezoelectric

**DOI:** 10.1038/s41467-022-32825-9

**Published:** 2022-08-29

**Authors:** Wei Feng, Bingcheng Luo, Shuaishuai Bian, Enke Tian, Zili Zhang, Ahmed Kursumovic, Judith L. MacManus-Driscoll, Xiaohui Wang, Longtu Li

**Affiliations:** 1 State Key Laboratory of New Ceramics and Fine Processing, School of Materials Science and Engineering, Tsinghua University, 100084 Beijing, China; 2 College of Science, China Agricultural University, 100083 Beijing, China; 3 School of Science, China University of Geosciences, 100083 Beijing, China; 4 Department of Materials Science and Metallurgy, University of Cambridge, Cambridge, CB3 0FS UK

**Keywords:** Materials science, Ferroelectrics and multiferroics

## Abstract

Piezoelectric materials provide high strain and large driving forces in actuators and can transform electrical energy into mechanical energy. Although they were discovered over 100 years ago, scientists are still searching for alternative lead-free piezoelectrics to reduce their environmental impact. Developing high-strain piezoelectric materials has been a long-term challenge, particularly challenging for the design of high-strain polycrystalline piezoelectrics containing no toxic lead element. In this work, we report one strategy to enhance the electrostrain via designing “heterostrain” through atomic-scale defect engineering and mesoscale domain engineering. We achieve an ultrahigh electrostrain of 2.3% at high temperature (220 °C) in lead-free polycrystalline ceramics, higher than all state-of-the-art piezoelectric materials, including lead-free and lead-based ceramics and single crystals. We demonstrate practical solutions for achieving high electrostrain in low-cost environmentally piezoelectric for various applications.

## Abstract (web summary)

Developing high-strain piezoelectric materials has been a long-term challenge. Here, the authors report one strategy to enhance the electrostrain via designing heterostrain through atomic-scale defect engineering and mesoscale domain engineering.

## Introduction

Piezoelectric materials are universal and important materials for a variety of applications, such as actuators and sensors. Piezoelectrics undergo strain in response to stimulation by an electric field and function in a responsive and controlled mode. Compared with other classes of materials that strain under the action of external physical stimulation, e.g. shape memory alloys^1^, electrorheological materials^2,3^, and magnetostrictive materials^4^, piezoelectric actuators have the advantage of fast response, good frequency characteristics, and resistance to electromagnetic interference. The benchmark electrostrain of 1.7% was reported in single crystals of Pb(Zn_1/3_Nb_2/3_)O_3_–PbTiO_3_ (PZN-PT)^5^. However, the growth of high-quality single crystals has many disadvantages such as high cost, complex and delicate control of experimental parameters, and harsh environments, which preclude practical applications of them^6^. In contrast to single crystal, developing non-textured polycrystalline piezoelectric consumes lower cost, shorter time, and less energy, providing a practical solution for large-scale applications. Unfortunately, the electrostrain of polycrystalline piezoceramics is much lower than that of single crystals. For instance, the maximum electrostrain reported in lead-free polycrystalline piezoceramic is below 0.7%^7^, while the maximum electrostrain reported in lead-based polycrystalline piezoceramic is 1.3%^8^. In addition, the lead element is restricted by the Restriction of Hazardous Substances (RoHS) Directive and the Waste Electrical and Electronic Equipment (WEEE) Directive due to its toxicity and damage to the environment. Even though the ban on lead in commercial electronic products is gradually being implemented, lead-based piezoelectric materials are still in an irreplaceable position in some highly sophisticated technologies and aerospace. Therefore, achieving larger electrostrain in piezoelectric materials, especially in lead-free materials, is a crucial requirement for the actuation application. Furthermore, in the last few decades there have been few advances in achieving large electrostrain at high temperatures (over 200 °C). For example, the maximum electrostrain at high temperature is below 0.6%^7^.

In principle, a large electrostrain can be achieved through multi-scale engineering of the composition-structure-property relationship. The contribution of electrostrain is divided into two parts: intrinsic contributions and extrinsic contributions. Intrinsic contributions originate from piezoelectric and electrostrictive effects. Extrinsic contributions come from the switching of non-180° domains and volume changes produced by the non-ferroelectric to ferroelectric phase change^9^. Generally, constructing a morphotropic phase boundary (MPB) can produce coupling of different polarization directions in different phases and thus significantly improve the small-signal piezoelectric response *d*_33_. However, an MPB does not normally show a large reverse switching of the ferroelectric domains or an improvement of the electrostrain. The introduction of defects can provide a restoring force for the reversible switching in non-180° domains and exhibit a huge recoverable strain of 0.75%^10^, but the formation of defects depends on the aging process. The stability of defects becomes the key point in influencing the strain stability, especially under the electric and thermal fields, which could irreversibly control oxygen vacancy concentration^11^. Constructing reversible electric field-induced phase transitions through ionic doping can increase the crystal symmetry^7,12,13^. Wu et al.^14^ combined field-induced phase transitions and charged point defects to improve electrostrain. But the symmetry of ferroelectric phases was improved, and the temperature stability of electrostrain was limited below a low ferroelectric-to-relaxor transition temperature (~127 °C). Hence, at higher temperatures (~200 °C), the increased symmetry of the ferroelectric phase hinders the phase transition induced by an electric field. Thus, with increasing temperature, there is a reduction in the electrical strain, which means the high-temperature actuation is worse^15,16^. Therefore, to overcome this problem and enhance the electrostrain, the design criterion needs to be to improve the contribution of reversible domain switching while maintaining low crystalline symmetry, thus enabling enhanced coupling of long- and short-range polarizations. In this work, we report one strategy to enhance the electrostrain via designing “heterostrain” through atomic-scale defect engineering and mesoscale domain engineering. We achieve an ultrahigh electrostrain of 2.3% at high temperature (220 °C) in lead-free polycrystalline ceramics, higher than all state-of-the-art piezoelectric materials, including lead-free and lead-based polycrystalline ceramics. The strong atomic hybridization, large ionic displacement, in-plane rotation of oxygen octahedra and energy level shifts induced by vacancies and electric fields, improve the stability of the disordered structure and give rise to heterogeneous domains, thus elucidating the mechanism for the large enhancement of electrostrain. We demonstrate practical solutions for achieving high electrostrain in low-cost environmentally piezoelectric for various applications.

**Fig. 1:**
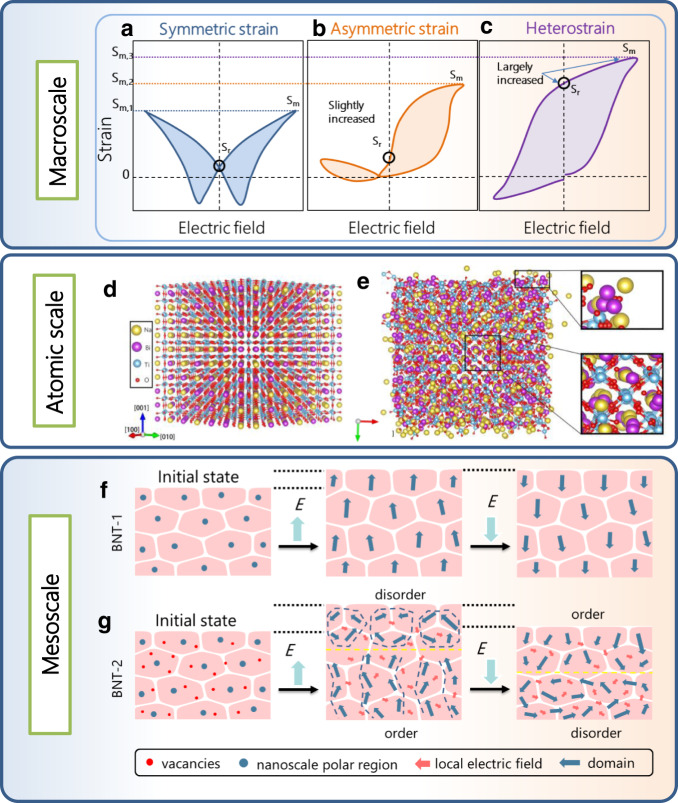
Design of piezoelectric with heterostrain. Comparison of macroscale bipolar strain curves for piezoelectric with **a** symmetric strain, **b** asymmetric strain, and **c** heterostrain. Both cases of symmetric and asymmetric strain have already been experimentally synthesized, while the piezoelectric with heterostrain was not done before this work. Ionic displacement at high temperature of BNT with disorder Bi/Na in a 7 × 7 × 10 supercell before (**d**) and after (**e**) 100,000 steps for 100 ps at 1400 K from large scale molecular dynamic simulation. Schematic ferroelectric domains of **f** BNT-1 and **g** BNT-2 ceramics.

## Results

### Macroscale design piezoelectric with heterostrain

Piezoelectric strain can be classified into three types: symmetric strain, asymmetric strain, and heterostrain. In general, most piezoelectrics display typical butterfly-shaped bipolar strain curves (Fig. 1a) with symmetric loops under the electric field, named “symmetric strain”. This type of piezoelectric has a very low residual strain (*S*_r_ close to zero) and low maximum value (*S*_m_ below 0.3%), which originates from a reversible transition between the classic ferroelectric phase and relaxor ferroelectric phase. In this case (Fig. 1a), the bipolar strain remains in the same direction under a change of direction of the electric field, and its values on both sides are close to each other. This phenomenon of symmetric strain has been widely demonstrated in piezoelectric compounds, such as lead zirconate titanate^17^, barium titanate, bismuth ferrite, potassium sodium niobate^18^, Pb(Mg_1/3_Nb_2/3_)O_3_-PbTiO_3_ (PMN-PT)^19,20^, Sr_0.35_Na_0.25_Bi_0.35_TiO_3_(SBNT)^21^, etc. When a considerable number of dopants are introduced in the Na_0.5_Bi_0.5_TiO_3_ (BNT) lattice, a strain curve with asymmetric loops (Fig. 1b) is discovered in the dopant-induced BNT-based piezoelectric ceramics. Here we name this type of piezoelectric strain “asymmetric strain”. In this case (Fig. 1b), one side of the strain increases, and the other decreases. Compared with symmetric strain, both S_m_ and S_r_ for asymmetric strain slightly increase. The phenomenon of asymmetric strain is proven in ions doped BNT-based ceramics, such as ((Bi_1/2_(Na_0.84_K_0.16_)_1/2_)_0.96_Sr_0.04_)(Ti_0.75_Nb_0.25_)O_3_ ceramics^7,14^. The *S*_m_ level is increased only slightly from 0.3% for symmetric strain to 0.7% for asymmetric strain, but the value is still very low. We note that in a normal ferroelectric like PZT^9^, there is only one stable strain state. In other words, whatever the value of the electrostrain, it will revert to the same strain state when the electric field is removed. Further increases in electrostrain with electric field are challenging because the strain in one direction is not built up upon cycling.

Here we report a phenomenon of “heterostrain” in BNT-based piezoelectric ceramics (Fig. 1c). We show that the strain along the negative direction of the electric field becomes negative, compared to it being positive for the symmetric and asymmetric strain cases. The positive and negative signs of the strain become consistent with the positive and negative signs of the electric field direction. *S*_m_ continues to increase as the electric field increases from negative to positive values. The large memory strain gives two stable strain states, a high strain state and a low-strain state, which can be switched to each other when changing the direction of the applied electric field. This result implies the contribution of domain wall motion and, therefore, we define these two strain states as heterostrain (Fig. 1c). Compared to symmetric and asymmetric strain, Sr is considerably increased. Therefore, higher *S*_m_ is expected in the case of a piezoelectric with heterostrain. Currently, both types of piezoelectric with symmetric and asymmetric strains have already been experimentally synthesized. However, ferroelectrics with heterostrain have neither been proposed or synthesized experimentally before. Herein, we achieve an ultrahigh electrostrain of 2.3% at high temperature (220 °C) via designing the “heterostrain” in BNT-based piezoelectric ceramics.

### Atomic-scale defect engineering and mesoscale-domain analysis

To achieve heterostrain in polycrystalline piezoelectric, we firstly conduct atomic-scale defect engineering and mesoscale-domain analysis. We simulated the sintering process from molecular dynamics methods (Fig. 1d, e). Large Bi or Na ionic displacements are observed on the surfaces after BNT annealing at 1400 K for 100 ps, while the Ti–O frameworks of [TiO_6_] octahedra almost remain constant in the inner supercell. Na and Bi_2_O_3_ dissociate from the supercell and Ti–O tetragons reconstructed on the surface, which inevitably produces A-site vacancies and oxygen vacancies. Experimental manipulation of vacancies is therefore key to enhancing electrical strain. Secondly, mesoscale domain engineering is employed to achieve the ultrahigh strain. Figure 1f, g show a mesoscale-domain analysis of piezoelectric ceramics under polarization. When an electric field is first applied, the nanoscale polar region of the rhombohedral phase in BNT increase to a distinct mesoscale domain, and the domain switches along the direction of the electric field. Macroscopically, the monoclinic phase transforms into a rhombohedral phase, and strain occurs in the direction of the electric field. When the electric field is removed, the strain along the polarization direction is maintained. It is reported that polycrystalline ferroelectric materials consists of a sequence of switching mechanisms: rapid movement of non-180° domain walls, the main switching phase with 180° and non-180° switching events, and creep-like non-180° domain wall movement^22^. When an opposite electric field is applied to the polarized sample, the 180° switching of the domains will no longer produce strain in the direction of the electric field due to its central symmetry. Only a small portion of the domain that is not 180°-switched will contribute to the strain. To enhance the contribution of domains, point defects such as oxygen and A-site vacancy are introduced to enhance the disorder of the local structure. It is reported that 180° switching in the BNT was realized through three intermediate steps of 71° ferroelastic switching upon electric field reversal^23^. When vacancy defects are introduced, the distortion of Ti–O octahedra is further enhanced. The formation of ordered and disordered regions and switching of 180° domains in BNT-2 are blocked and help us to construct multiple heterogeneous domain structures to achieve the switching between a polarization ordered state and disordered state (Fig. 1g). In BNT-2, the ordered and disordered regions both exist with different proportions, forming the multiple heterogeneous domain structures. Through the switching between a disordered and ordered polarization state, an enhanced electrostrain can be achieved.

### Fabrication of BNT piezoelectric ceramics

Based above atomic-scale defect engineering and mesoscale-domain analysis, we fabricate two types of BNT piezoelectric ceramics, BNT-1 and BNT-2. To inhibit the volatilization of Bi and Na, and thus to reduce the A-site vacancy and oxygen vacancy defects, the green bodies of BNT-1 are embedded in the mother powder and sintered in an alumina crucible with an alumina lid, as shown in Supplementary Fig. 1, In the case of the BNT-2, the Al_2_O_3_ lid is replaced by a ZrO_2_ lid with a porosity of 20%. The ZrO_2_ lid has holes inside so that the oxygen partial pressure can be engineered with different sintering temperatures to modulate the oxygen vacancy content. By using inductively coupled plasma-optical emission spectrometry (ICP-OES), the mole ratio of BNT-1 and BNT-2 samples was obtained as 0.498:0.474:1.000 and 0.496:0.468:1.000, respectively (Supplementary Table 1). The oxygen vacancy concentration in BNT-2 is 1.4 times than that of BNT-1. This is consistent with volatilization of Na being prevalent, while the loss of Bi is relatively small. Qualitative analysis of the molar ratios of the elements measured by ICP-OES indicates that the higher volatility of the A-site elements in BNT-2 leads to more oxygen vacancies.

### Piezoelectric performances of BNT polycrystalline ceramics

**Fig. 2:**
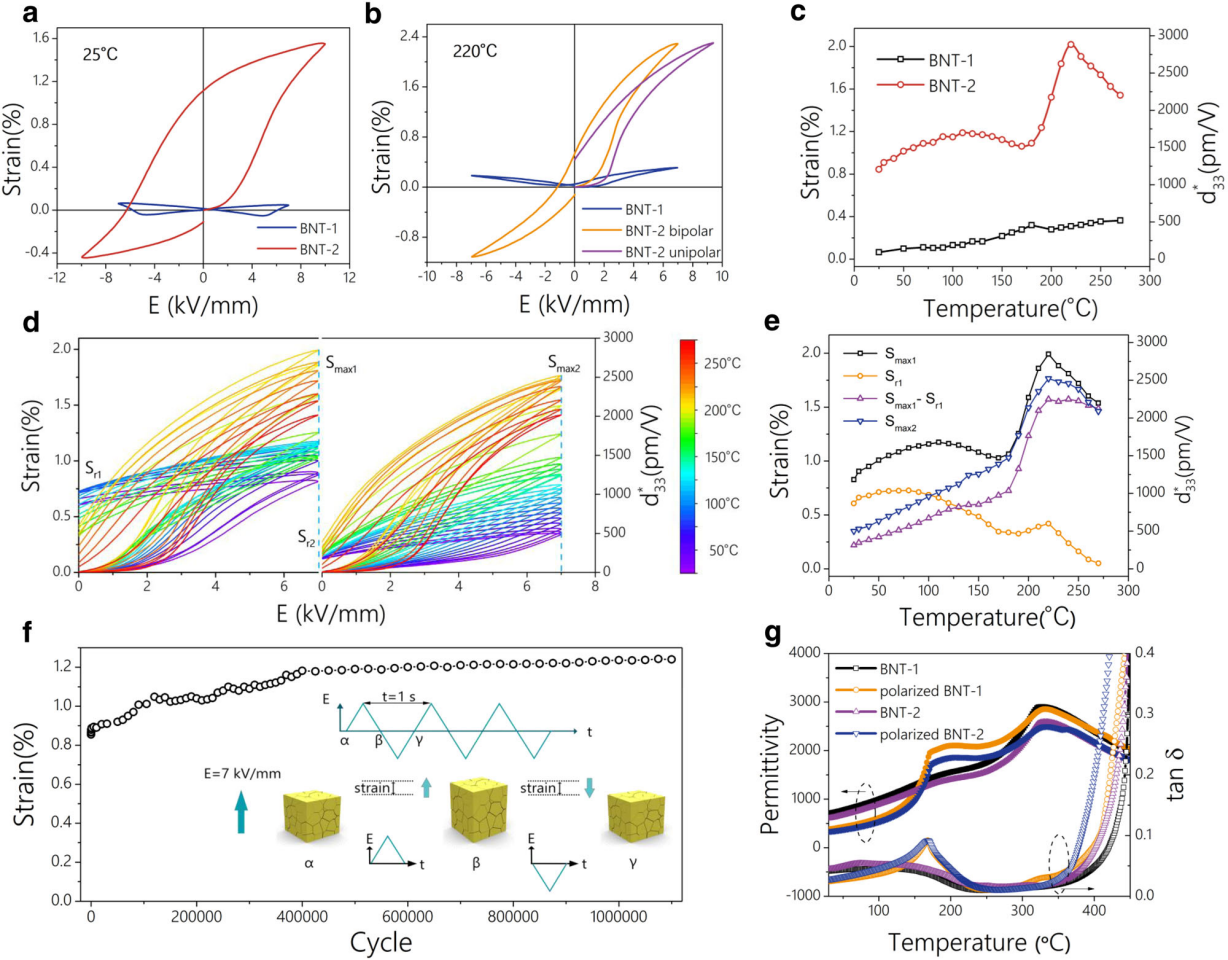
Piezoelectric performances of BNT polycrystalline. Strain curves of BNT-1 and BNT-2 samples at **a** 25 °C and **b** 220 °C measured at 1 Hz. **c** Temperature dependence of strain and *d*_33max_^*^ of BNT-1 and BNT-2 measured at 7 kV/mm and 1 Hz. **d** Two consecutive unipolar strain tests with increasing temperature. After each temperature test, the sample returns to its initial state with an equal amount of reverse electric field. **e** *S*_max1_, *S*_max2_, *S*_r1,_ and *S*_max1_−*S*_r1_ of BNT-2 obtained from **d** as the temperature is increased. **f** Cyclic stability from fatigue tests of BNT-2 at the electric field of 7 kV/mm (1 Hz). **g** Temperature dependence of dielectric constant (*ε*_r_) and loss (tan *δ*) for the BNT-1 and BNT-2 under 1 kHz.

We measured the electrostrain performance of lead-free BNT polycrystalline piezoelectrics (Fig. 2, Supplementary Figs. 2 and 3). The electrostrain of the BNT-2 sample is <1.5% at room temperature under an electric field of 10 kV/mm, which is significantly <0.12% of the BNT-1 samples. The memory strain is as high as 1.1%. The large electrostrain results in a high d_33_^*^ (the large-signal piezoelectric coefficient) of 1500 pm V^−1^. With increasing temperature, the electrostrain increases gradually (Fig. 2c). When the temperature rises above 180  °C, the strain increases sharply owing to the reversible phase transitions induced by the electric field. This is consistent with the depolarization temperature of BNT ceramics, as evidenced by the temperature-dependence dielectric property curves (Fig. 2g. and Supplementary Fig. 4). The strain profile of BNT-1 above 220 °C is strengthening as temperature increases (Supplementary Fig. 2), which is due to the enhancement of the internal bias field caused by the movement of oxygen vacancies under the combined effect of high temperature and electric field, resulting in the increase of its strain. In contrast, BNT-2 sample, which itself has a high degree of strain asymmetry, is degraded in strain due to its excess oxygen vacancies that change under the synergistic effect of high temperature and electric field. With increasing temperature, the Ti–O distance of both BNT-1 and BNT-2 remains almost unchanged, indicating the oxygen octahedra are stable during the increasing temperature process. For both BNT-1 and BNT-2, the Bi–O distance and Na–O distance show an obvious increase with increasing temperature, indicating the large ionic diffusion of Bi and Na among the neighboring octahedra gaps. The Na–O distance is larger than that of the Bi–O distance, revealing the larger ionic diffusion of Na than that of Bi. This originates from the hybridization of Bi 6p and O 2p states. However, the A-O bond length (the Bi–O bond) in BNT-2 is larger than in BNT-1 (Supplementary Fig. 5), showing that the Bi–O bond of BNT-2 is weakened, explaining the strain increase of BNT-2, which is also confirmed by Raman analysis. At a temperature higher than 220 °C, more oxygen vacancies are thermally activated at high temperatures, which increased the leakage current under the electric field, resulting in a lower strain at over 220 °C.

In addition, the d_33_^*^ also reaches the maximum of 3286 pm V^−1^ under an electric field of 7 kV/mm. Two consecutive unipolar strain tests were then performed as the temperature increased (Fig. 2d). After each temperature test, the sample is returned to its initial state under an equal amount of reverse electric field. In Fig. 2e, *S*_max1_ is the maximum strain of the unipolar strain in the first cycle and *S*_max2_ is in the second cycle. And *S*_r1_ is the residual strain after removal of the electric field in the first cycle. *S*_r1_ shows a decreasing trend with the increase of temperature, while the changes of *S*_max1_−*S*_r1_ and *S*_max2_ are almost the same, indicating that the high residual strain comes from the non-180° switching and stabilization of the domain, while the reversible strain can be achieved by applying a cyclic electric field.

**Fig. 3:**
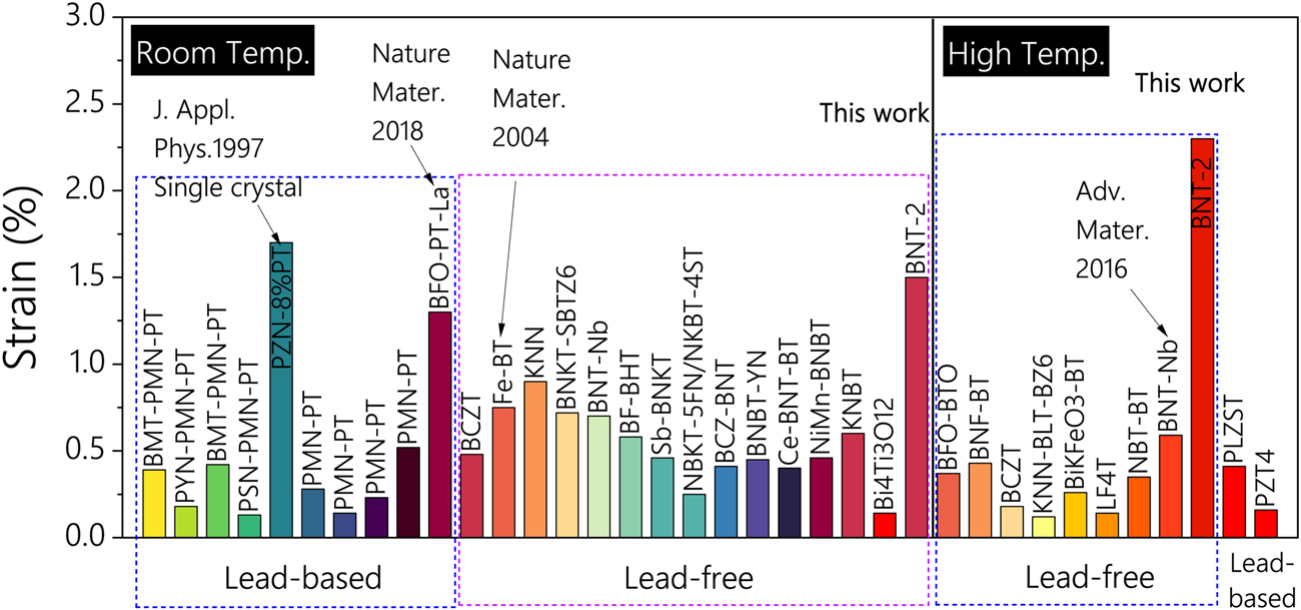
Maximum electrostrain of the state-of-the-art lead-based and lead-free polycrystalline piezoelectrics. The present work is noted as the red star in both room temperature and high-temperature regions. The 2.3% electrostrain at high temperature of present lead-free polycrystalline piezoelectrics is higher than state-of-the-art lead-based piezoelectrics, as listed in detail in Supplementary Table 2.

The maximum electrostrain of the state-of-the-art lead-based and lead-free polycrystalline piezoelectric is compared in Fig. 3 and listed in Supplementary Table 2. Designing the heterostrain enable the ultrahigh electrostrain to be achieved. At room temperature (25 °C), the obtained electrostrain of 1.5% is considerably higher than other piezoelectric materials (Fig. 3), including BMT-PMN-PT(0.42%)^24^, PMN-PT (0.52%)^25^, BFO-PT-La (1.3%)^8^, BT-Fe (0.75%)^10^, BCZT(0.48%)^26^, KNN(0.9%)^27^, BNKT-SBTZ (0.72%)^28^, BNT-Nb (0.7%)^7^, etc., reaching the benchmark electrostrain value of 1.7% in single crystals of Pb(Zn_1/3_Nb_2/3_)O_3_–PbTiO_3_ (PZN-PT)^5^. At high temperatures (220 °C), the bipolar strain and unipolar strain both reach extremely high values of 2.3% under electric fields of 7 kV/mm and 9.4 kV/mm, respectively. This is highest of all the reported piezoelectric materials, including lead-free and lead-based ceramics and single crystals, for instance, BNF-BT(0.34%)^29^, BFO-BTO(0.37%)^30^, BNT-Nb(0.59%)^7^, NBT-BT (0.35%)^31^, PZT (0.16%)^32^, PLZST(0.41%)^33^, etc.

Furthermore, changes in the phase signal φ_33_ and small-signal d_33_ were detected simultaneously upon straining (Supplementary Figs. 6–9). It can be found that the strain of the BNT-2 sample (Supplementary Fig. 6) is nonvolatile compared with that of the BNT-1 sample (Supplementary Fig. 7). A high and a low-strain state are observed in BNT-2 samples, which can be switched by controlling the direction of the electric field. Meanwhile, φ_33_ remained in −150° to −170° and *d*_33_ switches between −50 pm/V and −130 pm/V, indicating that the 180° polarization reversal does not exist in the BNT-2 sample. And it can lead to the disappearance of negative strain in the direction of the electric field, which is related to the strain originating mainly from the non-180° domain switching (Supplementary Fig. 10)^22,23^. Fortunately, the heterostrain phenomenon shown in Fig. 1a is synthesized in this work in the BNT-2 sample. Comparatively, BNT-1 sample is classic piezoelectric with symmetric strain as shown in Fig. 1a, which has a standard symmetrical butterfly curve with only one stable strain state (Supplementary Fig. 7). φ_33_ switches between 0° and −180° and *d*_33_ correspondingly switch symmetrically between 50 pm/V and −50 pm/V (Supplementary Fig. 9) during a 180 ° polarization reversal. Fatigue tests were carried out at the electric field of 7 kV/mm (1 Hz) at room temperature as shown in Fig. 2f. The electric field in the early stages played a similar role in the activation and the electrostrain increased from 0.85% to 1.24%. The strain remained unchanged and showed great stability during more than one million test cycles. The high-temperature fatigue also shows good stability at 150 °C (5 kV/mm, 1 Hz) in Supplementary Fig. 11. Until the 300,000 cycles, the strain is maintained at about 1.3%, showing high cyclic stability.

### Microstructure and morphology of the BNT ceramics

**Fig. 4:**
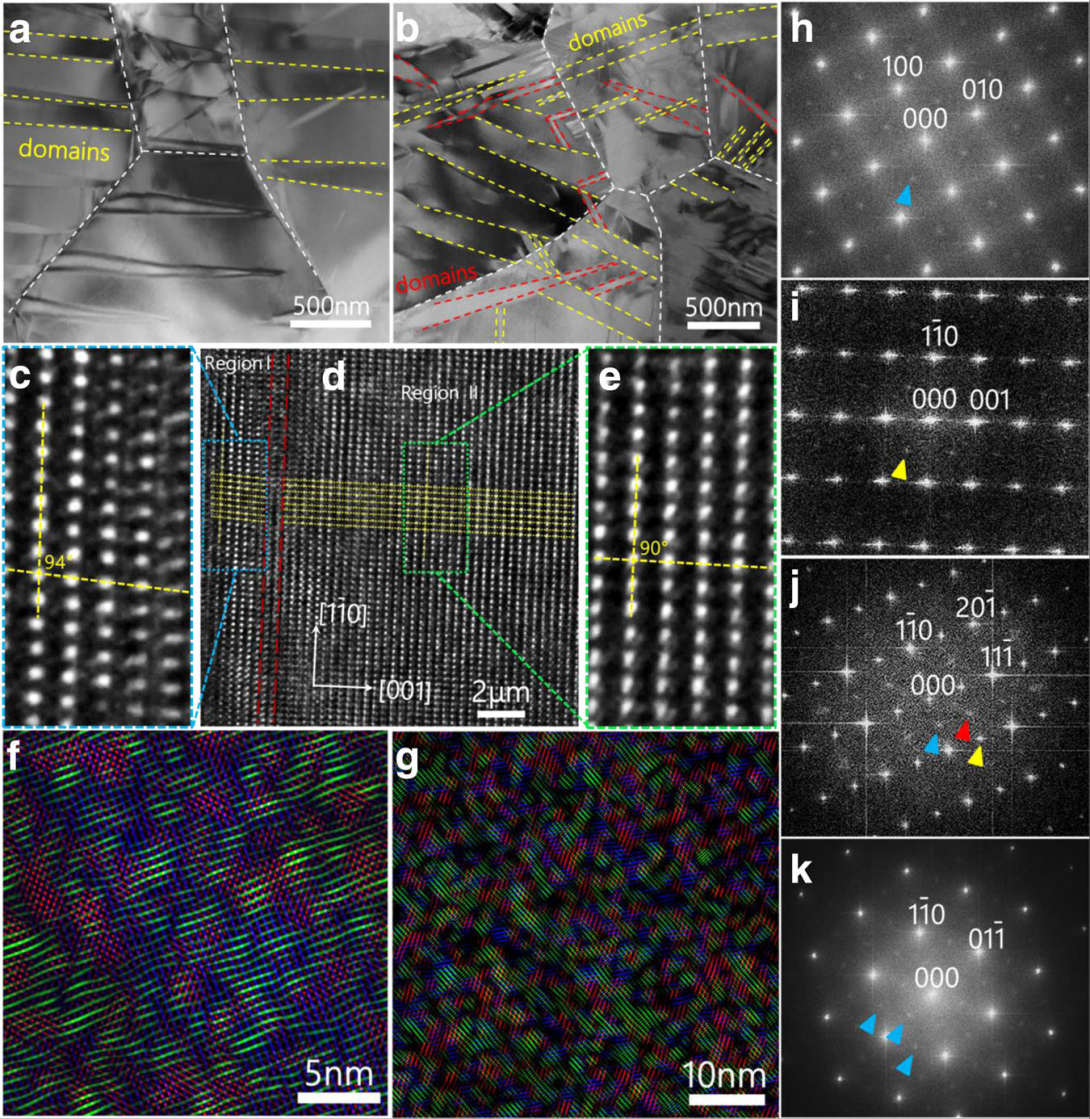
Microstructure of BNT polycrystalline. TEM images of **a** BNT-1 sample and **b** BNT-2 sample showing ferroelectric domains. **d** HRTEM images of polarized BNT-2 samples viewed down the [110] direction. **c** and **e** show magnified images of region I and region II, respectively. **f** HRTEM RGB image of polarized BNT-2 sample viewed along the [112] direction reconstructed from the three images obtained by filtering the original image using three diffraction spots visible in the FFT image (**h**). These three images are green, filtered using the diffuse intensity centered on ½{ooe}; blue, filtered using the lattice spots at ½{ooo}; and red, filtered using the lattice spots at ½{oee}. **g** HRTEM RGB image of polarized BNT-2 sample viewed down the [111] direction reconstructed from the three images obtained by filtering the original image using three sets of ½{ooe} spots, visible in the image FFT (**k**). The FFT of HRTEM along the **h** [001], **i** [110], **j** [112], and **k** [111] zone axis in polarized BNT-2 samples. Yellow arrows highlight ½{ooo} super diffraction spots that arise from an anti-phase rotation of the oxygen octahedra and give rise to rhombohedral symmetry. Blue arrows highlight ½{ooe} super diffraction spots and blue arrows represent the in-phase rotation of the short-range tetragonal distortion regions.

To further underline the mechanism of ultrahigh electrostrain for BNT-2, we characterize the microstructure and morphology of the BNT samples using transmission electron microscopy (TEM) and high-resolution TEM (HRTEM) as shown in Fig. 4, Supplementary Figs. 12 and 13. BNT-1 sample with a higher ordered structure has an obvious ferroelectric stripe domain (Fig. 4a). In contrast, BNT-2 samples exhibit a more complex disordered domain structure (Fig. 4b). The BNT-2 samples are composed of domains of varying sizes, and the average domain size is smaller than that of the BNT-1 samples (Supplementary Fig. 13). Strong coupling between neighboring grains is observed from the cross-grain boundaries of the domains.

From Fig. 4c–e, the crystal orientations of [110] in region I (Fig. 4c) and region II (Fig. 4e) show around 4° torsion along the [001] direction. Thus, the angle between the crystal planes of [110] and [001] in region-II is 90° while that in region-I is about 94°, showing one feature of monoclinic symmetry for BNT-2, which is due to the change of the ion displacement polarization direction and the distortion of [TiO_6_] octahedra, evidenced by the first-principles calculations. From the corresponding fast Fourier transform (FFT) (Fig. 4i), the diffraction pattern splits and elongates along the [001] direction, which is due to the stress caused by the lattice mismatch between the two different regions. The local structure of the BNT is generally considered to be rhombohedral and accompanied by an anti-phase tilt of [TiO_6_] octahedra. However, due to a reduction of Bi–O bonds, there is an additional in-phase tilted local structure^34^. As a result, the refined XRD results of BNT-2 show an average monoclinic Cc structure^35,36^. Similar to the monoclinic structure of the polar nanoregions (PNRs) reported in lead-based materials, this monoclinic symmetry is due to the combination of different polarization directions in the PNRs, which are usually small in size and subject to inhomogeneous local polar structures^37^. However, the increase of vacancies in BNT-2 makes it more difficult for BNT to form long-range order with rhombohedral symmetry on a global scale.

**Fig. 5:**
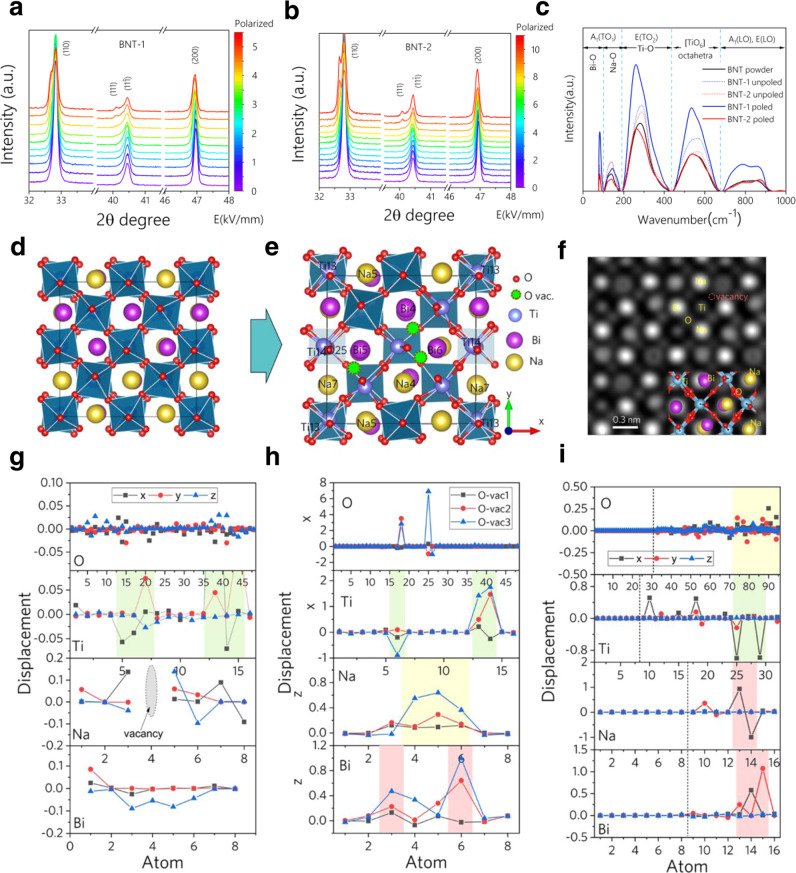
Mechanism of ultrahigh electrostrain of BNT polycrystalline. In-situ XRD under the DC electric field of **a** BNT-1 sample and **b** BNT-2 sample. **c** Raman spectra of different samples. The relaxed crystal structure of BNT with **d** zero and **e** 3/48 oxygen vacancies. **f** Simulated HAADF STEM image of BNT containing 3/48 oxygen vacancies. **g** The effect of Na vacancy on the ionic displacement of Bi, Na, Ti, and O atoms of BNT samples with 1/8 Na vacancy. **h** The relative relaxation ratio induced by oxygen vacancies of BNT with various amounts (1/48, 2/48, 3/48) of oxygen vacancies. **i** The relative relaxation ratio induced by the electric field of 0.01 V/Å for BNT containing 1/48 oxygen vacancy. The left region of the dashed line is the inner layer and the right region of the dashed line is the surface layer.

In BNT-2, ½(20$\bar{1}$) diffraction spots marked by the red arrows in Fig. 4j indicate the emergence of a new distorted order of oxygen octahedra^38^. The ½{oee} super diffraction is only observed in the orthorhombic intermediate phase in the evolution of the BNT structure formed at high temperatures (200–300 °C)^39^. The competition between tetragonal and rhombohedral symmetries leads to an intermediate state of ion displacement polarization, verified by the previous local monoclinic symmetry. From Fig. 4j, orthorhombic distortion nano-regions (red) and tetragonal distortion regions (green) are dispersed in the rhombohedral (blue) matrix. Generally, the ions in polarized BNT such as BNT-1 relax to adapt to the long-range ordered rhombohedral and the local tetragonal distortions with the tilt of the in-phase octahedra being drastically reduced^34^. In BNT-2, however, the tetragonal distortion is maintained and orthorhombic distortion occurs. This is associated with an increase in the phase transition barrier caused by an increase of A-site and oxygen vacancies, which is consistent with the in-situ XRD analysis (Fig. 5b). In addition, three ½{ooe} super diffraction spots in Fig. 4k are selected to reconstruct the RGB HRTEM image. It can be seen that three distorted nano-regions are uniformly distributed throughout the region (Fig. 4 g), which is the same as the unpolarized BNT reported in the previous work^40^.

As a result, the BNT-1 sample only shows one unique rhombohedral phase feature. For example, only a rhombohedral lattice is observed along all the [001], [110], [111], and [112] zone axes. The BNT-2 sample exhibits a complex structure with multiphase coexistence. For example, a monoclinic phase character is observed in the local structure along the [110] zone axis; three types of structures, namely rhombohedral, orthorhombic, and tetragonal are observed along the [112] zone axis; tetragonal phase is observed along [001] and [111] zone axes. The complex structure with multiphase coexistence including monoclinic, rhombohedral, orthorhombic and tetragonal phases is therefore linked to the presence of a high electrostrain.

### Relationship between macroscale properties and the microstructure

To further figure out the relationship between macrostructure and microstructure, we performed in-situ XRD with temperature-dependence, in-situ XRD with electric-field-dependence, XPS, and Raman spectroscopy. The grain sizes of BNT-1 and BNT-2 from SEM are similar (Supplementary Fig. 14) and the XRD results show no obvious difference for both samples (Supplementary Fig. 15). Analysis of in-situ XRD with temperature-dependence for BNT-2 at 20–300 °C shows that the residual polarization decreases with increasing temperature, and the electrostrain contribution from the reversible phase transitions between the tetragonal and rhombohedral phases increases^7,41^, enabling the maximum strain to be maintained at the higher temperature of 220 °C (Supplementary Fig. 16). A different structure evolution for BNT-1 and BNT-2 is observed by in-situ XRD with electric-field-dependence (Fig. 5a, b). For instance when the electric field increases to ~4.5 kV/mm, the (110) and (111) peaks of the BNT-1 sample split due to the electric field-induced phase change from monoclinic to rhombohedral phase^42^. In the case of the BNT-2 sample, the same phase transition occurred when the electric field increases to about twice that of the BNT-1 sample (~9 kV/mm) revealing that the BNT-2 structure is more stable under the external electric field. XPS analysis shows a decrease in the intensity of Ti 2*p* and O 1 *s* in polarized BNT-2 compared to polarized BNT-1, accompanied by a separation toward higher binding energies (Supplementary Fig. 17). The peak at around 531 eV was attributed to the O^2−^ in the oxygen-deficient region. BNT-2 has a larger proportion of O 1 *s* peak at 531 eV than BNT-1, which indicates that a higher content of non-lattice oxygen and oxygen vacancies in the BNT-2 than BNT-1 samples and the appearance of Ti^4+^ cations with higher binding energy^43,44^.

Raman results are shown in Fig. 5c and the peaks representing the vibrational modes of Ti–O, Bi–O, and Na–O are fitted (Supplementary Fig. 18 and Supplementary Table 3). Before polarization, the Raman spectra of BNT-1 and BNT-2 are basically the same, and similar to that of disordered BNT precursor powder. Specifically, Ti–O modes at 200–400 cm^−1^ are split into two peaks: one peak ~280 ^cm−1^ representing the rhombohedral contribution and the peak ~390 cm^−1^ representing the tetragonal distortion^45^. After polarization, the contribution of tetragonal distortion increases for both BNT-1 and BNT-2 compared with samples before polarization. Raman peaks of BNT-1 become much narrower and sharper, with greater intensity and significantly enhanced order, while the Raman spectra of BNT-2 show broadened peaks, weakened intensity and less ordered structure. For example, two peaks for Ti–O modes of BNT-2 are centered at 260 ^cm−1^ and 360 cm^−1^, while that of BNT-1 are centered at 255 cm^−1^ and 315 cm^−1^. All the vibrational peaks in the polarized BNT-2 sample exhibit a larger full width at half maxima(FWHM) than that of BNT-1 due to disorder and to the dynamics of polar clusters^46^ (Supplementary Table 3). The broadening peaks reflects the fact that their ferroelectric order is weaker than that of BNT-1. In addition, the vibrational peaks of the Bi–O bonds showed no significant changes, indicating that the original structure of the polarized BNT-2 was maintained under the action of the electric field. Combined with first-principles calculations in Fig. 5i, it is observed that with an increase in the electric field, the ion displacement is greater and the Bi/Na distribution is more disordered. Therefore, it can be concluded that BNT-2 with more bismuth and oxygen vacancy defects has greater deformation and larger local structural disorder compared to the BNT-1 which maintains a locally disordered structure and is difficult to form as a long-range ordered rhombohedral phase upon application of an electric field.

### Electronic and atomic origin of the ultrahigh strain

We perform first-principles calculations to investigate the effects of sodium vacancies, oxygen vacancies, bismuth vacancies, and order/disordered Bi/Na distributions on the electronic and structural properties of BNT, as shown in Fig. 5 and Supplementary Figs. 19–25. The compositional disordered inhomogeneity of BNT is visually confirmed by using an aberration-corrected STEM technique^47^. Based on the experimental results, a series of BNT models with 1/48, 2/48, and 3/48 quantitative oxygen vacancies, are modeled based on a disordered Bi/Na structure. The high-angle annular dark-field imaging (HAADF) scanning transmission electron microscope (STEM) image is simulated as shown in Fig. 5f. Given the (001) plane, the appearance of oxygen vacancies reduces the intensity of denoting oxygen atoms. The ferroelectric polarization originates from the octahedral rotations and ionic displacements. The relative relaxation ratio induced by vacancies is expressed as Δ*z* = (*z*_i_−*z*_0_)/*z*_0_ × 100%, where *z*_i_ is the coordinate of structures containing vacancies, *z*_0_ is the coordinate of the structure without vacancies. Δ*z* denotes the relative relaxation ratio along [001] direction, and similarly, Δ*x* and Δ*y* denote the relative relaxation ratio along [100] and [010] directions (Fig. 5g–i, Supplementary Fig. 20, and Supplementary Tables 4–9). Positive values are ion relaxation along the positive direction and negative values are ion relaxation along the negative direction. Firstly, the effects of Na vacancies on the structure and ionic displacements of BNT ferroelectrics as shown in Fig. 5g. The presence of Na vacancy-induced ionic displacement value of Na is large than that of Bi ions. The effects of Na vacancy on the O displacement are tiny compared with that of Bi and Na ions. Secondly, from Fig. 5h, the existence of oxygen vacancies induces large ionic relaxation, shown in the shaded areas, of surrounding ions such as O-25, Ti-13, Na-5, and Bi-6 in Fig. 5e. Furthermore, the large ionic displacement of Bi ions occurs mainly in *z*-axis orientation, while the *x*- and *y*-axis ionic displacements remain almost unchanged (Supplementary Fig. 20A). Ti and O ionic displacements occur mainly along the *x*-axis while the *z*-axis ionic displacement remains almost unchanged (Supplementary Fig. 20C). More oxygen vacancies, larger ionic displacement. It can be concluded that the introduction of oxygen vacancies induces the in-plane rotation of octahedra and the vertical movement of A-site ions in the adjacent octahedral space. Comparing Fig. 5g, h, it can be seen that the effect of oxygen vacancy on [TiO_6_] octahedra is stronger than that of Na vacancy. The enhancement of the octahedral distortion in the BNT-2 sample can be confirmed by the TEM results (Fig. 4). Thirdly, the effects of the external electric field on the BNT surfaces are calculated (Fig. 5i and Supplementary Fig. 21). It is seen that the relative ionic displacement of all ions induced by the electric field occurs mainly in the surface layers along the *x*- and *y*-axes, i.e., in the (001) plane in Fig. 5d, while the relative ionic displacement along the *z-*axis remains nearly constant, clarifying that the application of an external electric field induces the in-phase tilt of [TiO_6_] octahedra. The corresponding {ooe} lattice spots still exist in the polarized BNT-2 samples. And the competition of two kinds of torsion, in-phase and anti-phase, leads to an intermediate state of ion displacement polarization as evidenced by the emergence of the orthorhombic symmetry, as well as the {oee} lattice spots.

The temperature effects of the strain/polarization are shown in Supplementary Fig. 22. With the temperature increasing from 0 K to 600 K, the value of Ti ionic displacement increases gradually along the [001] direction. The value of Ti ionic displacement along [001] is much larger than that along [100] and [010] directions. The increasing displacement along the [001] direction cannot enhance the distortion of the octahedra, which plays the opposite role to the electric field. Analysis of total density of states showed the bandgap of disordered BNT is similar to that of ordered BNT with a bandgap difference of 0.2 eV (Supplementary Fig. 23). For instance, the HSE06 band gaps of disordered Bi/Na structure and ordered Bi/Na structure are 3.7 eV and 3.9 eV, respectively. With increasing oxygen vacancies, the valence band maximum (VBM) shifts to a higher energy level, while the conduction band minimum (CBM) remains constant, resulting in a reduced bandgap. The HSE06 band gaps of BNT with (1/48, 2/48, 3/48) oxygen vacancies are 3.0 eV, 2.82 eV, and 2.58 eV. In contrast, the introduction of bismuth vacancies in BNT increases the bandgap to 4.5 eV compared to oxygen vacancies. The increase in oxygen vacancies can also be verified by the sudden increase in loss corresponding to the decrease in temperature in Fig.2 g. Hybridization between Ti 3*d*, Bi 6*p*, and O 2*p* states in the valence band and conduction band can be observed in Supplementary Fig. 24. Upon introduction of the oxygen vacancies, the electron loss of Ti ions induces a decrease in the level of CBM, as evidenced by the shift of Ti 3*d* states. For the case of 3/48 oxygen vacancies, the intermediate states originate from the hybridization among Ti 3*d*, Bi 6*p*, and O 2*p* states, and appear between the conduction band and valence band. Hybridization between Bi 6*p* and O 2*p* states is observed in the partial density of states, while the A-site Na ions do not contribute to CBM and VBM. Moreover, the introduction of Bi vacancies boosts the CBM to a higher energy level. Therefore, the hybridization of the Bi–O bond and the distortion of the octahedra have synergistic effects on the structure of BNT. In addition, it should be noted that due to the atomic number of the model, the model with more defects is used to analyze the local structure, it cannot represent the average structure of the whole sample. Therefore, the diversity enhancement of local octahedra distortions increases the threshold of the phase transition of the whole sample, further resulting in multiple heterogeneous domains. The large enhancement of electrostrain obtained in the BNT-2 material is caused by domain switching.

## Discussion

Generally, excessive volatilization of Bi elements will lead to the formation of TiO_2_ when the BNT material is sintered at high temperatures according to the results of molecular dynamics simulation and literature reports^48^. Therefore, to further explore the role of the TiO_2_, we conduct controlled experiments with multiphase ceramics composed of BNT and TiO_2_, as shown in Supplementary Discussion and Supplementary Figs. 26–28. During the sintering process, titanium oxide with a different stoichiometric ratio to Ti:O = 1:2 may be formed due to the lack of oxygen or the diffusion of Na ions. Its semiconductor nature ensures that it can provide charges to form a field. TiO_2_ is the additive, thus most of the TiO_2_ grains are located at the boundaries between BNT grains and the field will influence the local electric fields at the interface between titanium oxide and BNT, and further influence the domain distribution (Supplementary Fig. 26). The asymmetric strain curve similar to BNT-2 can be seen in Supplementary Fig. 26, but the electrostrain is less than half as large as BNT-2. On the other hand, it can be further verified that the large electrostrain of BNT-2 is not caused by TiO_2_ due to excessive volatilization of elements, but the structural disorder caused by the formation of appropriate defects in BNT.

Our results suggest that the ion doping method commonly used to construct electrically induced phase transformations in BNT is not the most effective method to boost the strain. In general, ion doping limits the distortion of the octahedra and fails to maintain the low symmetry of the ferroelectric phase. Although reversible strain can be improved by lowering the depolarization temperature to room temperature, the strain performance at high temperatures is killed. However, a moderate number of vacancies can maintain the stability of the disordered structure in BNT, enhance the distortion disorder of the oxygen octahedra, thus maintaining the low symmetry and further forming the heterogeneous domains. Strong coupling of long-range and short-range polarization, vacancies, and electric field-induced large ionic displacement, octahedra rotation, and energy level should allow for the ultrahigh electrostrain.

Besides having an ultrahigh electrostrain, BNT-2 ceramics exhibit a significant strain memory effect owing to two stable strain states at room temperature. As shown in Supplementary Figs. 29 and  30, the transition from low-strain state α to high-strain state β is achieved by a forward monopole input electric field, and a reverse monopole reset electric field can transform the high-strain state β into a low-strain state γ. The states α and γ have a similar low-strain response. Based on this strain memory effect, applications can be extended to memory, sensors, processors, transistors, etc. Analysis of the hysteresis and strain curves at different electric field, shows that continuous modulation up to a maximum displacement of 3 μm (strain of 1.11%) can be achieved under different electric fields and remains stable during multiple cycles, exhibiting a stable strain memory effect.

In conclusion, we proposed to achieve ultrahigh electrostrain via design heterostrain in piezoelectric ceramics based on atomic-scale defect engineering and meso-scale domain engineering. We achieved the electrostrain of 2.3% at 220 °C, higher than the state-of-the-art piezoelectric materials, including lead-free and lead-based polycrystalline ceramics and single crystals. Combined with the experiments with multiscale simulation, it is found that the large ionic displacement and octahedra distortions induced by vacancies and electric fields, give rise to multiple heterogeneous domains, thus elucidating the mechanism for the large enhancement of electrostrain. Based on this proof-of-concept, various applications including memories, sensors, processors, and transistors can be expected.

## Methods

### Fabrication of polycrystalline piezoelectric

Conventional solid-state reaction was used to prepare Bi_0.5_Na_0.5_TiO_3_ using Bi_2_O_3_ (≥99.9% purity), Na_2_CO_3_ (≥99.9% purity) and TiO_2_ (≥99.99% purity, rutile). Raw powders were dried at 200 °C for 12 h in a vacuum drying oven to remove adsorption water and crystal water. Then the raw powders were weighted according to their stoichiometric formula and ball-milled for 12 h in a nylon jar with zirconia balls as the medium. The slurries were dried at 70 °C and the size of the powder should be less than 60 mesh. The powders were calcined for 4 h at 850 °C and passed the 60 mesh again. The calcined powders were uniaxially pressed into pellets of 10 mm in diameter, and the green bodies were sintered at 1100 − 1160 °C for 2 h. It should be noted that the difference lies in, BNT-1 which is embedded in the mother powder and sintered in an alumina crucible with an alumina lid to reduce volatilization and defects. In the case of the BNT-2, the Al_2_O_3_ lid is replaced by a ZrO_2_ lid. The ZrO_2_ lid has a porosity of 20% so that the oxygen partial pressure can be changed to influence oxygen vacancy content. There is no concern about any possible reaction between BNT and containers because more than 5 green pellets are stacked, and the bottom and top samples are not used for subsequent analysis. And multiple sintering was carried out under controlled conditions to ensure the reproducibility of the sample. The scheme of sintering setups is shown in Supplementary Fig. 1.

Multiphase ceramics composed of BNT and TiO_2_ were sintered for a comparative experiment. The prepared BNT powders and the commercial TiO_2_ nanoparticles with the size of 25 nm were weighed according to the weight ratio of 100:1. The dried slurries were uniaxially pressed into pellets of 10 mm in diameter, and the green bodies were put in covered alumina crucibles sintered at 1120–1140 °C for 1 h with a rapidly increasing and decreasing temperature rate of 30 °C min^−1^_._ The relatively short sintering time and rapid increasing/decreasing ramp rate were adopted to prevent the diffusion between TiO_2_ and BNT and to suppress the growth of TiO_2_ nanoparticles.

### Measurement of piezoelectric performances

Prior to electrical measurements, the sample pellets were polished and coated with a silver paste, followed by the burnt at 400 °C to form the electrodes. The diameter of samples is 8.25~8.30 mm and the thickness is 0.3–0.4 mm. The ferroelectric hysteresis loops and strain curves were measured in silicone oil using a piezoelectric evaluation system (aixPES-TF Analyzer 2000E: mixes, aixACCT Systems GmbH, Aachen, Germany). Dielectric spectra and Impedance spectrum of samples were measured using an impedance analyzer (E4980A; Agilent Technologies Inc., CA, USA).

### Characterization

The crystal structures were characterized by X-ray diffraction (XRD, SmartLab9KW, Rigaku, Tokyo, Japan) with Cu Kα radiation. The in-situ XRD test was performed under the electric field. As the surface of sintered ceramic pellets was sputtered to form a gold film with an appropriate thickness to prevent X-rays from being absorbed by the electrodes. The in-situ XRD at high temperature was characterized by Bruker D8 Advance (Bruker AXS, Germany). Field emission scanning electron microscopy (FESEM, Merlin, ZEISS, Germany) was carried out to examine the surface of the as-sintered ceramic pellets. The microstructure is further analyzed using transmission electron microscopy (TEM, FEI Tecnai Talos F200). X-ray photoelectron spectroscopy (XPS) was characterized using an X-ray photoelectron microprobe (Escalab 250Xi, ThermoFisher, Britain) equipped with a standard monochromatic Al Kα excitation source (hν = 1361 eV). The Raman spectrum was measured by the HR800 (France, HORIBA Jobin Yvon). Inductively coupled plasma-optical emission spectrometry (ICP-OES) was measured by iCAP 6300 (Thermo Fisher Scientific, Leicestershire, UK).

### First-principles calculations

The effects of oxygen vacancy, bismuth vacancy, and the order/disorder Bi/Na distribution on the electronic and structural properties of BNT were calculated within first-principles calculations based on the density functional theory (DFT) framework as implemented VASP code^49–51^. The pseudopotentials were constructed by the electron configurations as Na 2p^6^3s^1^ states, Ti 3s^2^sp^6^3d^2^4s^2^ states, Bi 5d^10^6s^2^6p^3^ states, and O 2s^2^2p^4^ states. In all the calculations, the energy tolerance was 1 × 10^−8^ eV/atom and Hellmann−Feynman force tolerance is 0.01 eV/Å. The generalized gradient approximation (GGA) with the PBEsol exchange-correlation functional was used. HSE06 hybrid functional is used to improve the band gaps. A plane-wave basis kinetic energy cutoff of 600 eV and Gamma centered 2 × 2 × 3 k-points are adopted^52^. Postprocess of vacancies induced structural and electronic changes are analyzed via our homemade codes and represented using VESTA software^53^.

### Molecular dynamic simulation

Large-scale molecular dynamic simulations were performed using the large-scale atomic/molecular massively parallel simulator (LAMMPS) and MedeA environment^54^. A system of BNT with Bi/Na disorder distribution was constructed based on the supercell of BNT, which contains 4900 atoms. Buckingham style forcefield for the description of inorganic crystal structures such as perovskite, pyrochlore, and spinel structures was employed^55^. The dynamic process is performed with the NVT ensemble and Nosé-Hoover thermostat at the temperature of 1400 K. The number of steps is 100,000 for NVT-MD simulation and the time step is 1.0 fs, resulting in the total simulation time of 100 ps. The open visualization tool OVITO^56^ and VESTA^53^ is used for postprocessing and visualization.

## Supplementary information

Supplementary Information

## Data Availability

The datasets generated during and/or analyzed during the current study are available from the corresponding author on reasonable request.
