# Supplementary material for: Heterostrain-enabled ultrahigh electrostrain in lead-free piezoelectric
Source: Nat Commun. 2022 Aug 29;13:5086. doi: 10.1038/s41467-022-32825-9 (PMC9424301; doi:10.1038/s41467-022-32825-9)
Supplement: Supplementary file 1 — Supplementary Information [file 41467_2022_32825_MOESM1_ESM.pdf]

Supplementary Information for

Heterostrain-enabled ultrahigh electrostrain in lead-free piezoelectric

Wei Feng [1]†, Bingcheng Luo[2]*†, Shuaishuai Bian[1], Enke Tian[3], Zili Zhang[3], Ahmed Kursumovic[4], Judith L. MacManus-Driscoll[4], Xiaohui Wang[1]*, Longtu Li[1]

Affiliations:

[1]State Key Laboratory of New Ceramics and Fine Processing, School of Materials Science and Engineering, Tsinghua University, Beijing 100084, China.

[2]College of Science, China Agricultural University, Beijing 100083, China.

[3]School of Science, China University of Geosciences, Beijing 100083, China.

[4]Department of Materials Science and Metallurgy, University of Cambridge, Cambridge, CB3 0FS, UK.

*Corresponding author. Email: luobc21@cau.edu.cn and wxh@tsinghua.edu.cn

†These authors contributed equally to this work.

Supplementary Discussion

From the XRD (Supplementary Fig. 15) and SEM (Supplementary Fig. 14C) results, BNT and TiO2 are well combined. The addition of TiO_2 does not affect the symmetry of BNT with temperature shown as the dielectric temperature curve, but the corresponding temperature of loss sudden increase move to low-temperature obviously (Supplementary Fig. 27), which is due to the lower activation energy of oxygen vacancy at high temperature. The morphology of the domain was observed by TEM (Supplementary Fig. 26). It was found that the radially shaped domains centered on the TiO_2 grain, and the domain size at the grain boundary was small. Moreover, the domain was also interwoven like that in BNT-2. It indicates that the existence of TiO_2 influences the local electric fields at the interface between titanium oxide and BNT, and further influences the domain distributions near the TiO_2 grains. The asymmetric strain curve can be seen in (Supplementary Fig. 28). The strain of BNT-TiO_2 is between BNT-1 and BNT-2. Compared with BNT-2, the decrease of strain may be related to the distribution of TiO_2 in the ceramics, which depends on whether the grains of TiO_2 and BNT can be well sintered together. Moreover, the low compact ceramics will also lead to leakage conduction and electric breakdown, and a decrease of strain. The results of this control experiment show that the appropriate defects in BNT-2 will lead to more obvious structural disorders and affect the growth of domains. Excessive defects will lead to the formation of TiO_2. The large electrostrain of BNT-2 is not caused by TiO_2 due to excessive volatilization of elements but the structural disorder caused by the formation of appropriate defects in BNT.

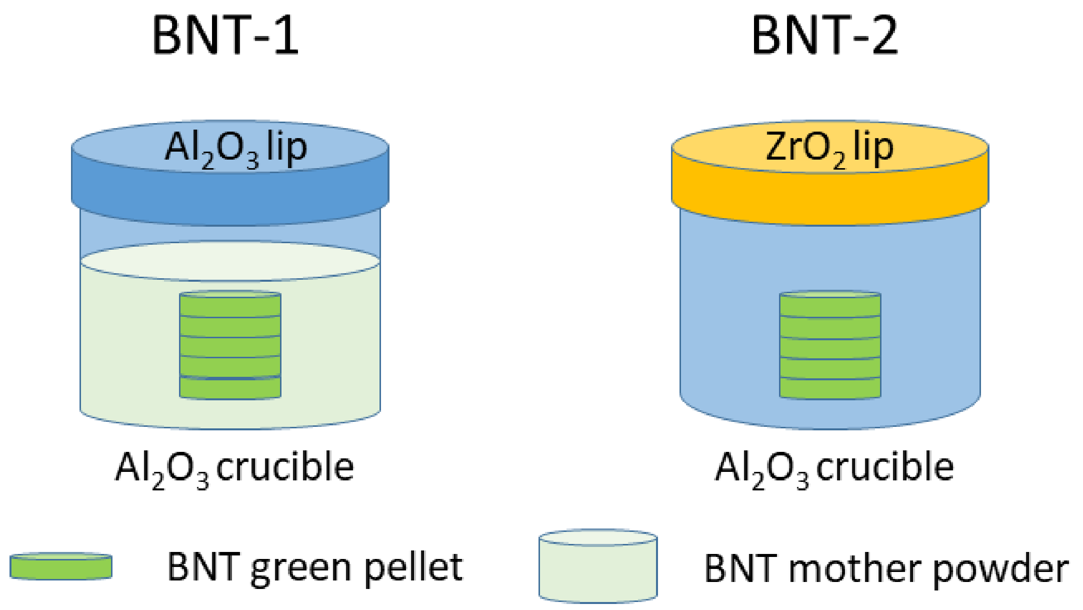

Supplementary Fig. 1. Scheme of experimental sintering setups. To inhibit the volatilization of Bi and Na, and thus to reduce the A-site vacancy and oxygen vacancy defects, the green bodies of BNT-1 are embedded in the mother powder and sintered in an alumina crucible with an alumina lid. In the case of the BNT-2, the Al_2O_3 lid is replaced by a ZrO_2 lid with a porosity of 20%.

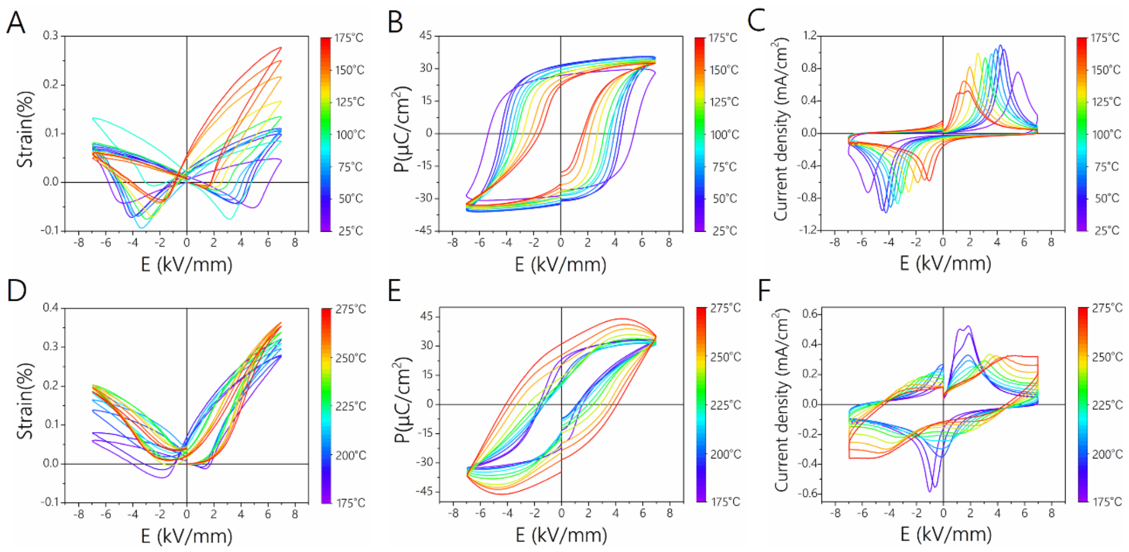

Supplementary Fig. 2. Piezoelectric performances. (A) and (D) Bipolar strain curves, (B) and (E) P-E hysteresis loops, (C), and (F) I-E curves of BNT-1 at different temperatures.

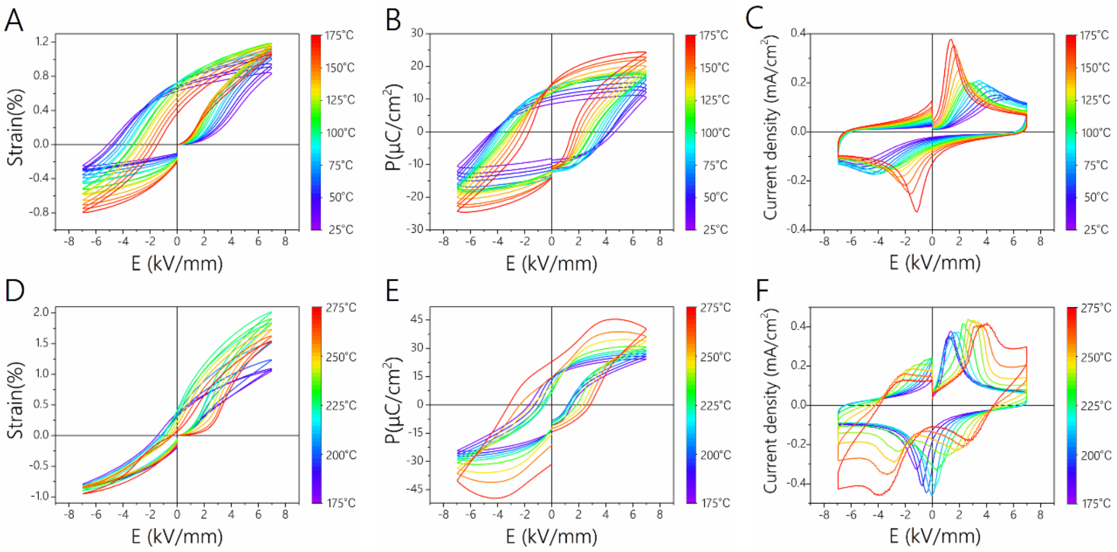

Supplementary Fig. 3. Piezoelectric performances. (A) and (D) Bipolar strain curves, (B) and (E) P-E hysteresis loops, (C), and (F) I-E curves of BNT-2 at different temperatures.

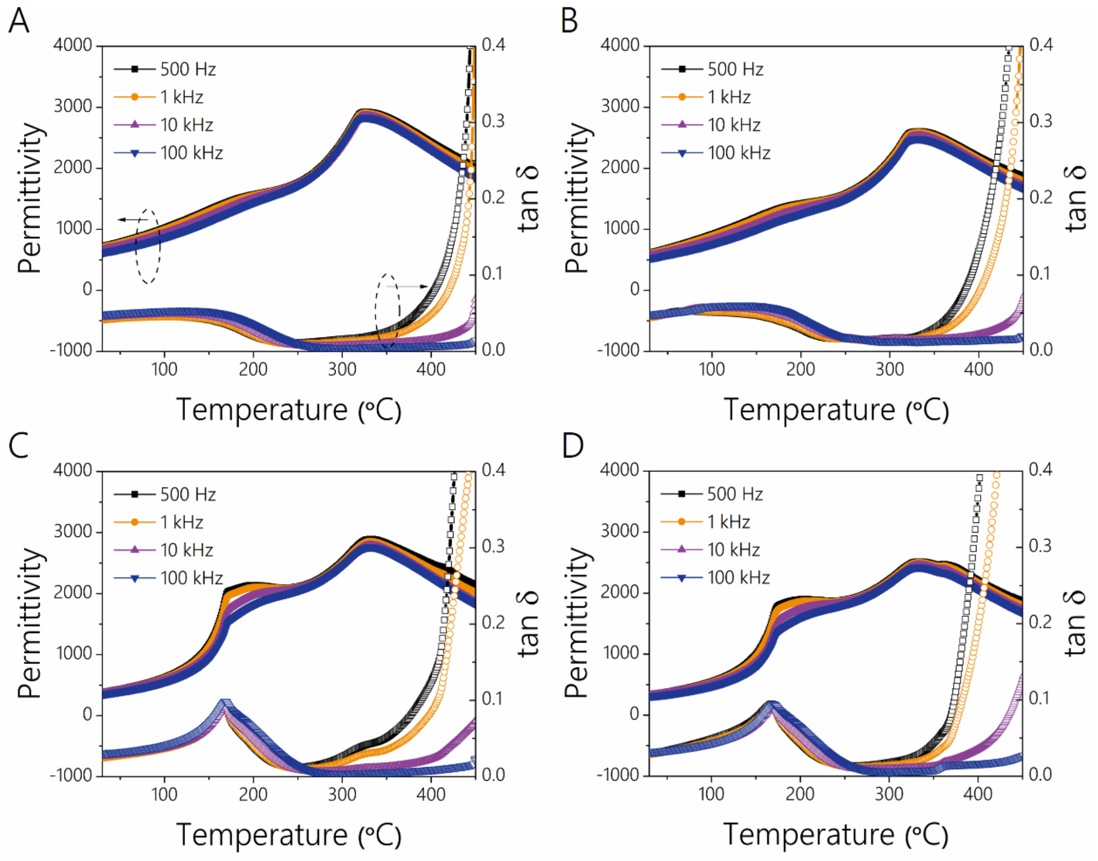

Supplementary Fig. 4. Temperature dependence of dielectric constant (εr) and loss (tan δ) of BNT samples under different frequency. (A) BNT-1, (B) BNT-2, (C) polarized BNT-1, and (D) polarized BNT-2 samples

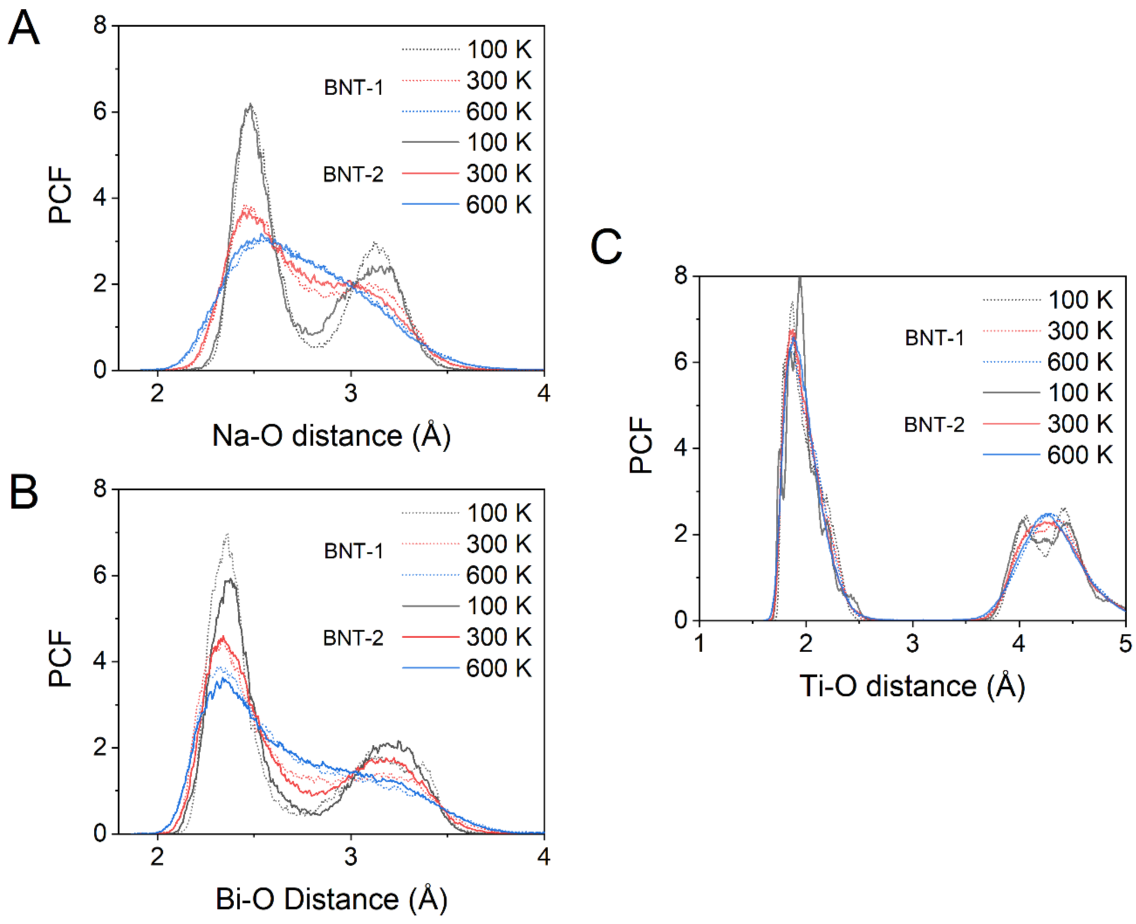

Supplementary Fig. 5. Ab initio MD simulation of A-O and B-O bond of BNT-1 and BNT-2. The pair correlation function of (A) Bi-O bond, (B) Na-O bond, (C) Ti-O bond at the temperature of 100 K, 300 K, and 600 K. Ti-O distance remains almost unchanged, indicating the oxygen octahedra maintains stable during the increasing temperature process. The Bi-O distance and Na-O distance show an obvious increase with increasing temperature, indicating the large ionic diffusion of Bi and Na among the neighboring octahedra gaps. The Na-O distance is larger than that of Bi-O distance, revealing the larger free degree of ionic diffusion of Na than that of Bi. This is originated from the hybridization of Bi 6p and O 2p states.

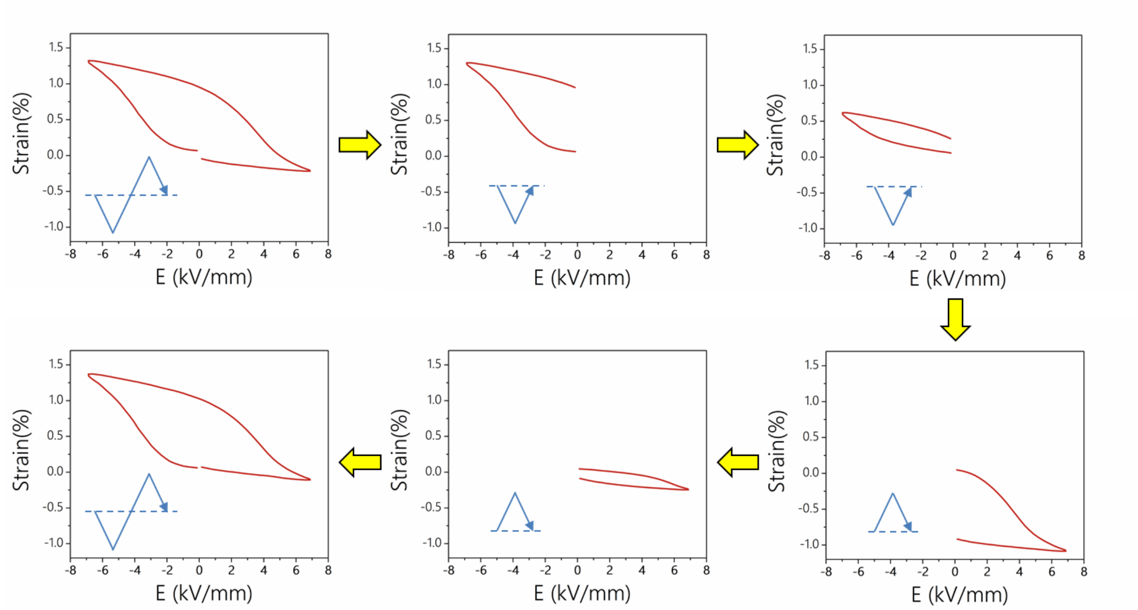

Supplementary Fig. 6. Bipolar strain curves of BNT-2 under continuously different waveform electric field. A high and a low strain state are observed, which can be switched by controlling the direction of the electric field.

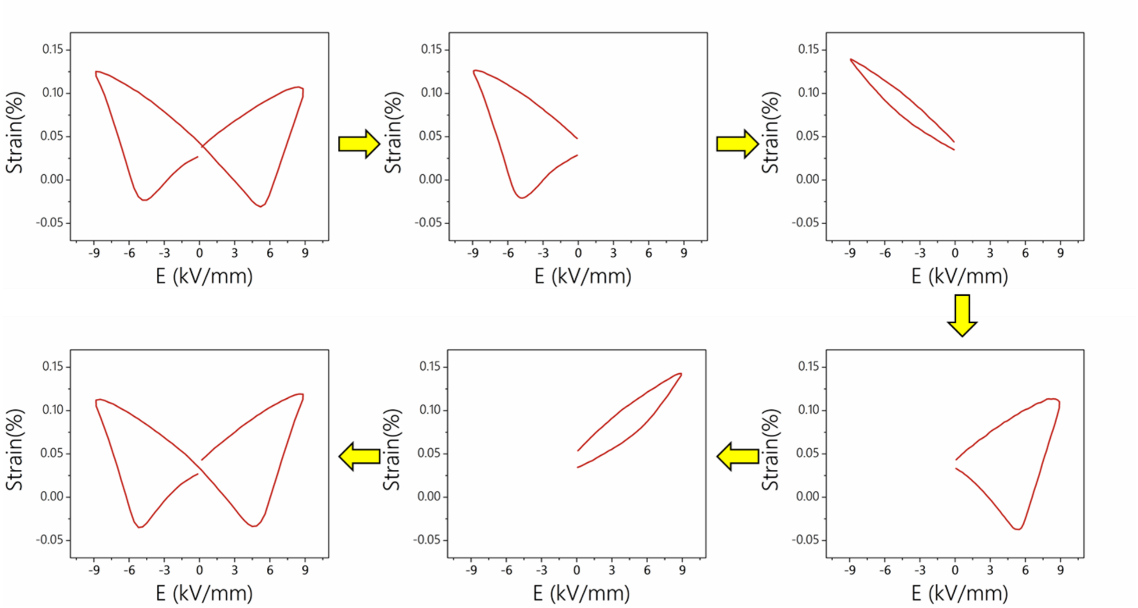

Supplementary Fig. 7. Bipolar strain curves of BNT-1 under continuously different waveform electric field. The waveform is the same as that in Supplementary Fig. 6.

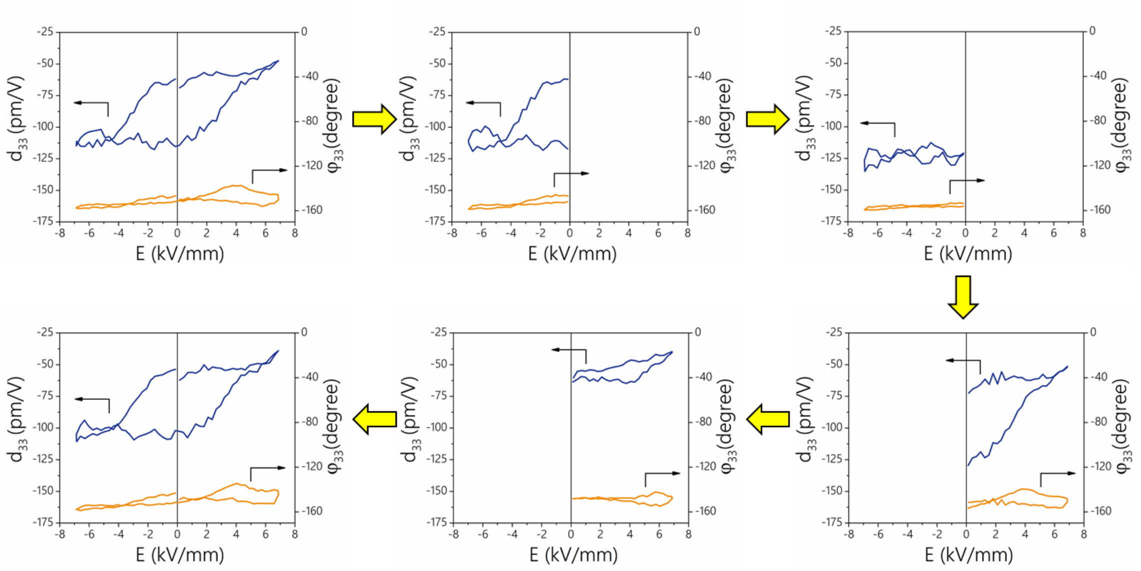

Supplementary Fig. 8. Phase signal φ33 and small-signal d33 of BNT-2 under continuously different waveform electric field. The waveform is the same as that in Supplementary Fig. 6.

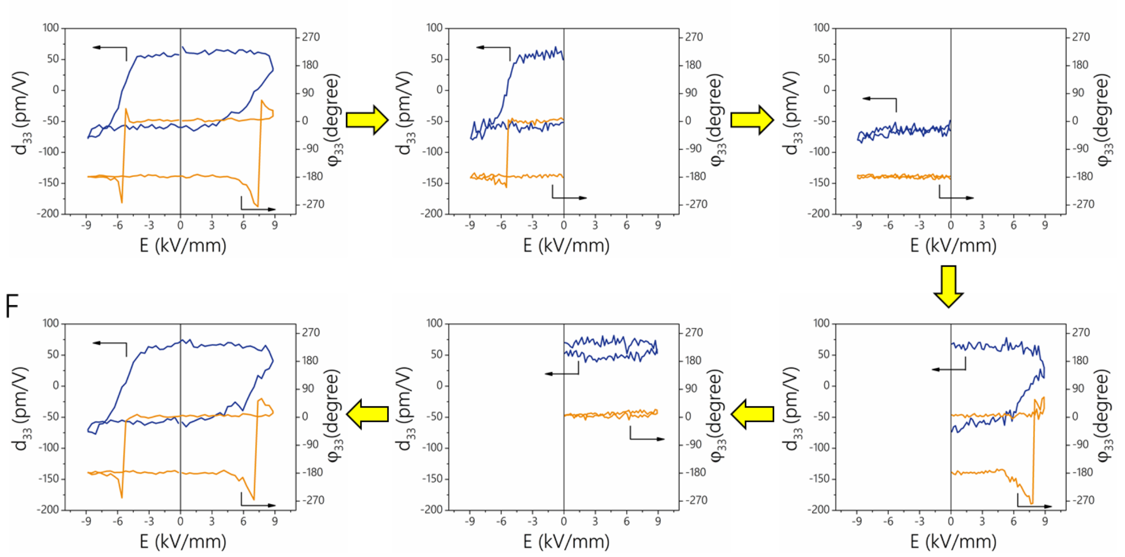

Supplementary Fig. 9. Phase signal φ33 and small-signal d33 of BNT-1 under continuously different waveform electric field. The waveform is the same as that in Supplementary Fig. 6.

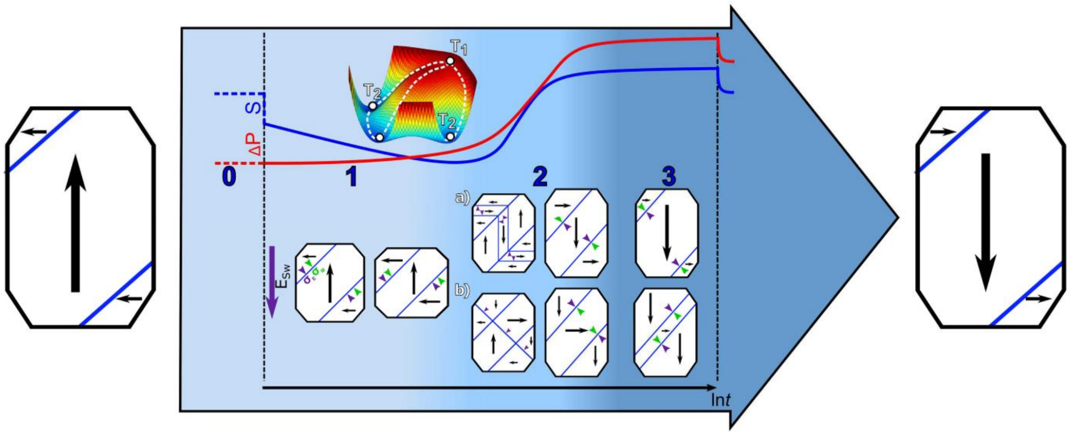

Supplementary Fig. 10 Schematic diagram of dynamic response of poling in $Pb(Zr,Ti)O_3$ polycrystalline ferroelectric materials. The three distinguishable regimes of the dynamic response are highlighted: 0) poled state, 1) initial non-180° domain wall movement, 2) main-switching phase by a) nucleation and sidewise growth of 180° domains and b) 180° polarization reversal by a nucleated non-180° domain occurring by synchronized non-180° reversal in grain, and 3) creep-like domain wall movement. The BNT-2 sample owns two stable polarization intensities, like in (a) or (b) and 3. (Referred to Acta Materialia, 2018, 157, 355-363)

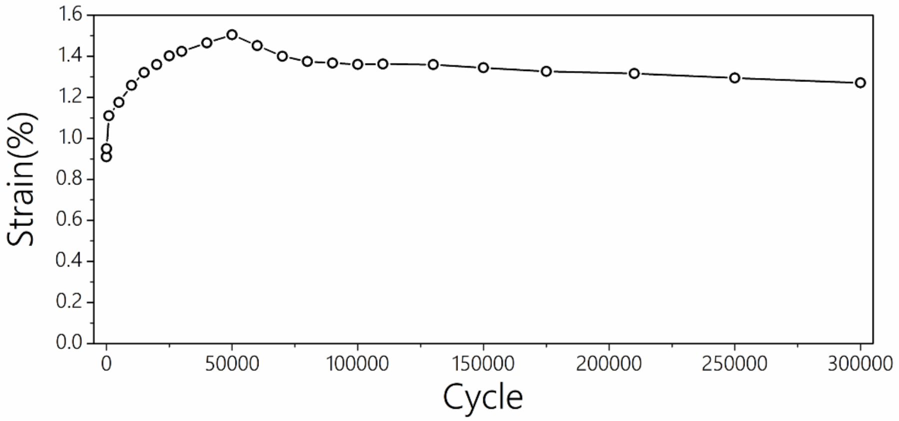

Supplementary Fig. 11. Cyclic stability from fatigue tests of BNT-2. Measurements are performed at the electric field of 5 kV/mm (1 Hz) and 150℃.

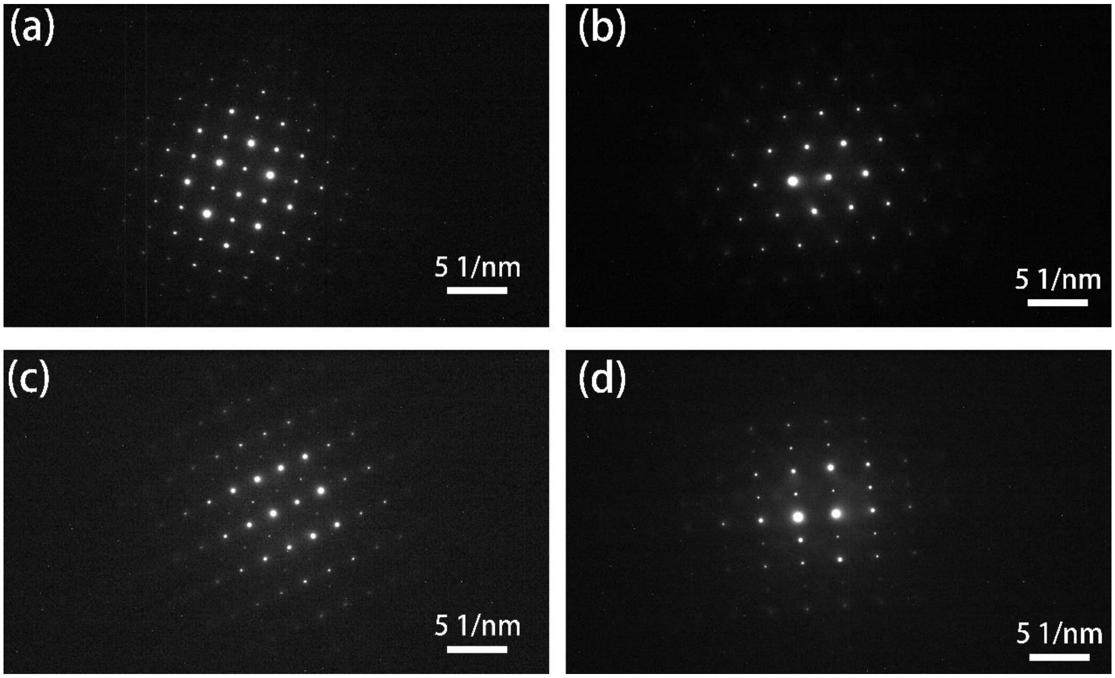

Supplementary Fig. 12. TEM electron diffraction patterns of BNT-1 along different directions. (a) [001], (b) [110], (c) [111], (d) [112]. In BNT-1, only a rhombohedral structure is observed along all the [001],[110], [111], and [112] zone axes.

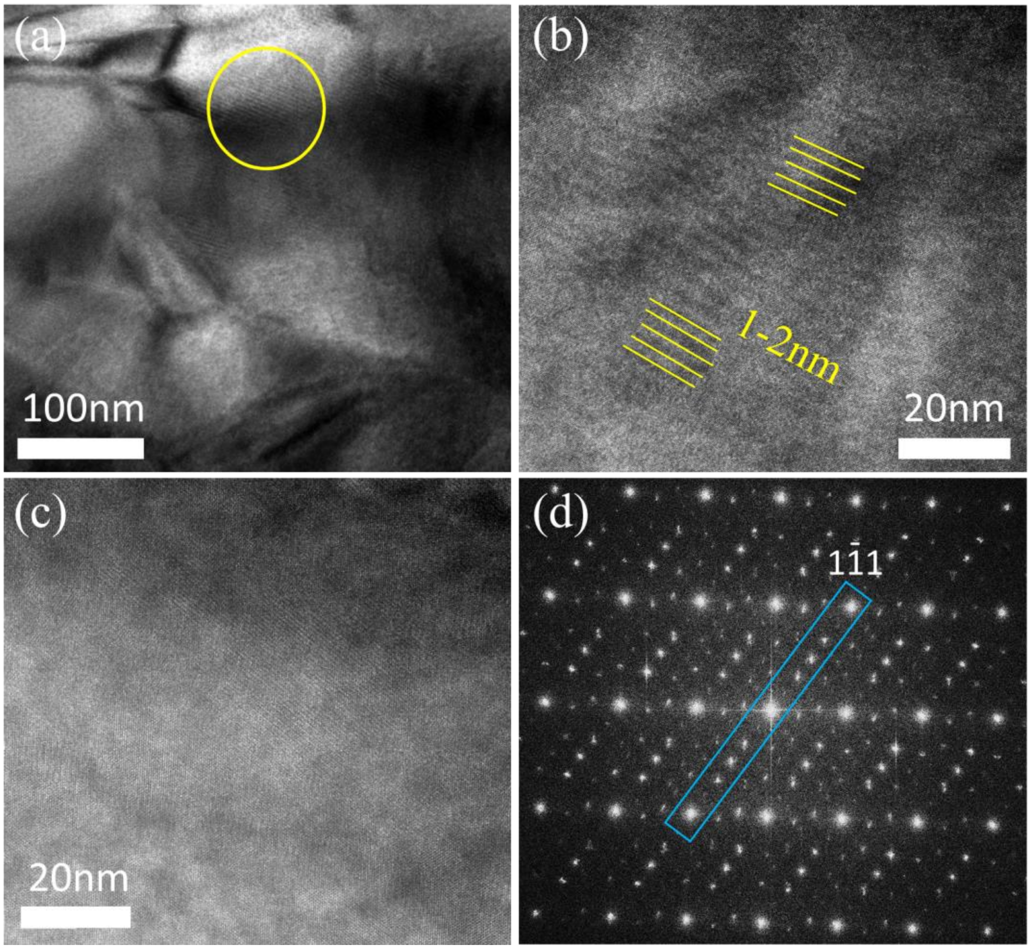

Supplementary Fig. 13. TEM images of BNT-2 ceramics. (a) nano-sized ferroelectric domains in BNT-2 ceramics, (b) enlarged view of the circle region in Fig. (a), (c) bright-field image of the [110] spatial axis, (d) FFT of Fig. (c)

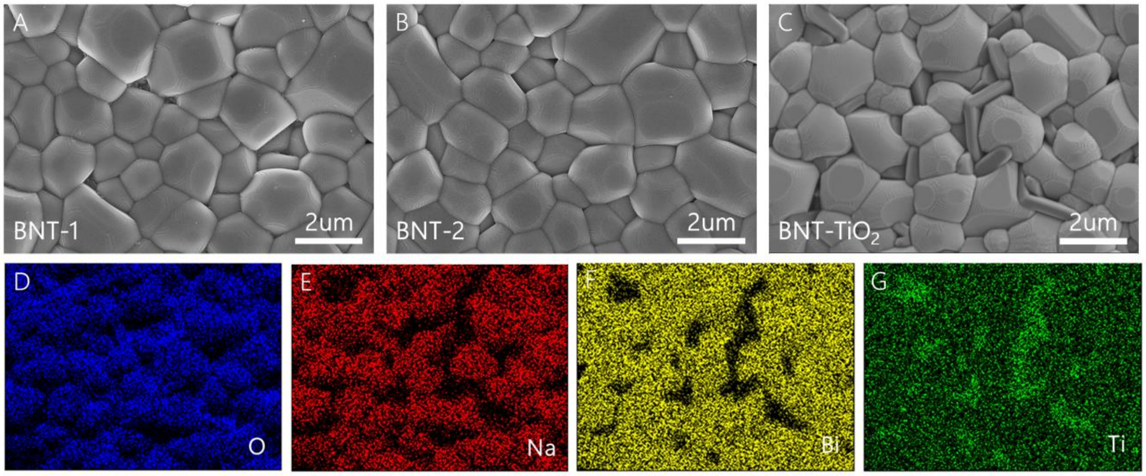

Supplementary Fig. 14. Surface SEM images of BNT samples. (A) BNT-1, (B) BNT-2, and (C) BNT-TiO2. SEM-EDS elemental mapping of (D) O, (E) Na, (F) Bi, and (G) Ti of BNT-TiO2.

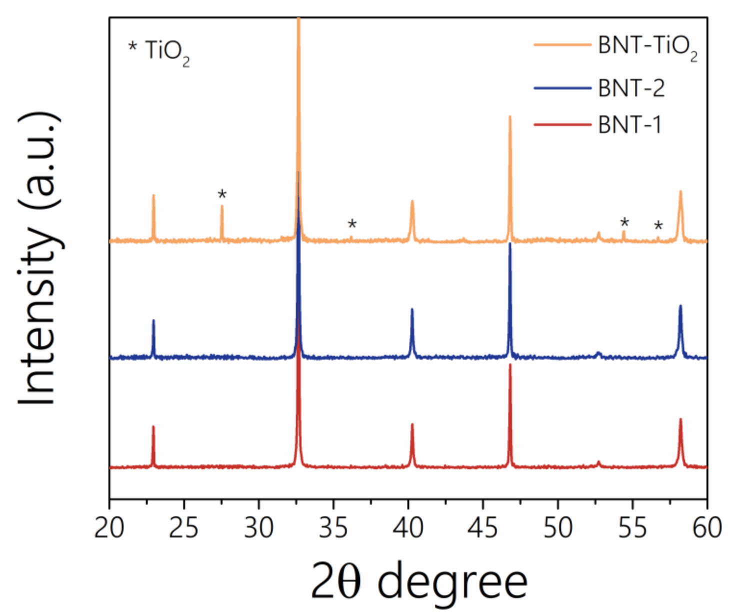

Supplementary Fig. 15. **X-ray diffraction patterns** of the BNT-1, BNT-2, and BNT-TiO2.

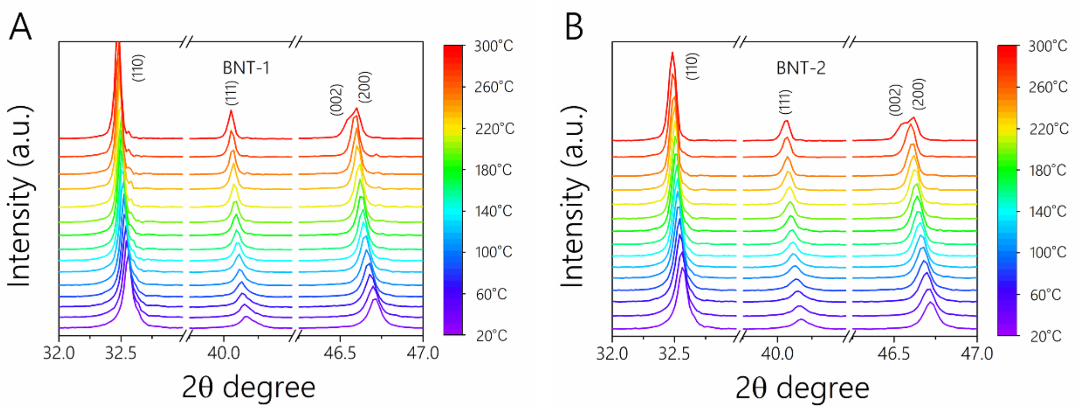

Supplementary Fig. 16. In-situ temperature-dependent XRD of BNT-1 (A) and BNT-2 (B) at 20-300°C. Both two samples have the same phase transition process with increasing temperature. With increasing temperature, only the peak shift occurs, and the (002) peak splitting begins near 300°C, which proves that the samples enter into tetragonal phase. With the increase of temperature and the decrease of remnant polarization, the electrostrain contribution of the reversible phase transition will increase, so the maximum electric strain will be obtained at a higher temperature of 220°C, which comes from the synergistic effect of defect bias field and reversible phase transition.

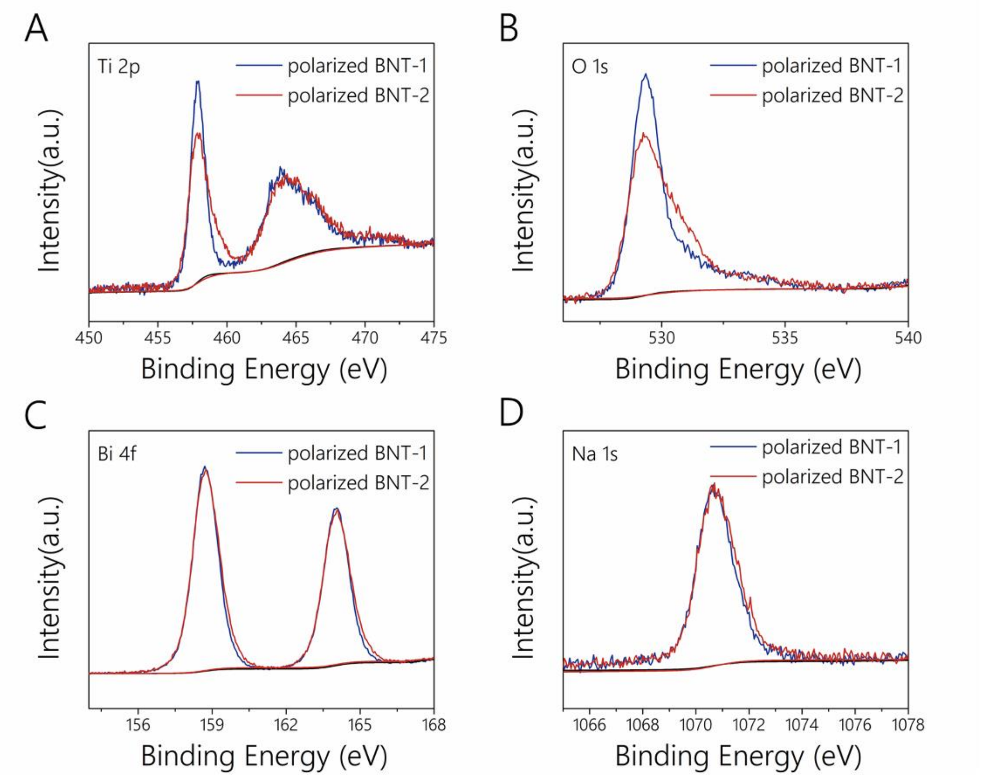

Supplementary Fig. 17. XPS spectra BNT samples. (A) Ti 2*p*, (B) O 1*s*, (C) Bi 4*f* and (D) Na 1*s*.

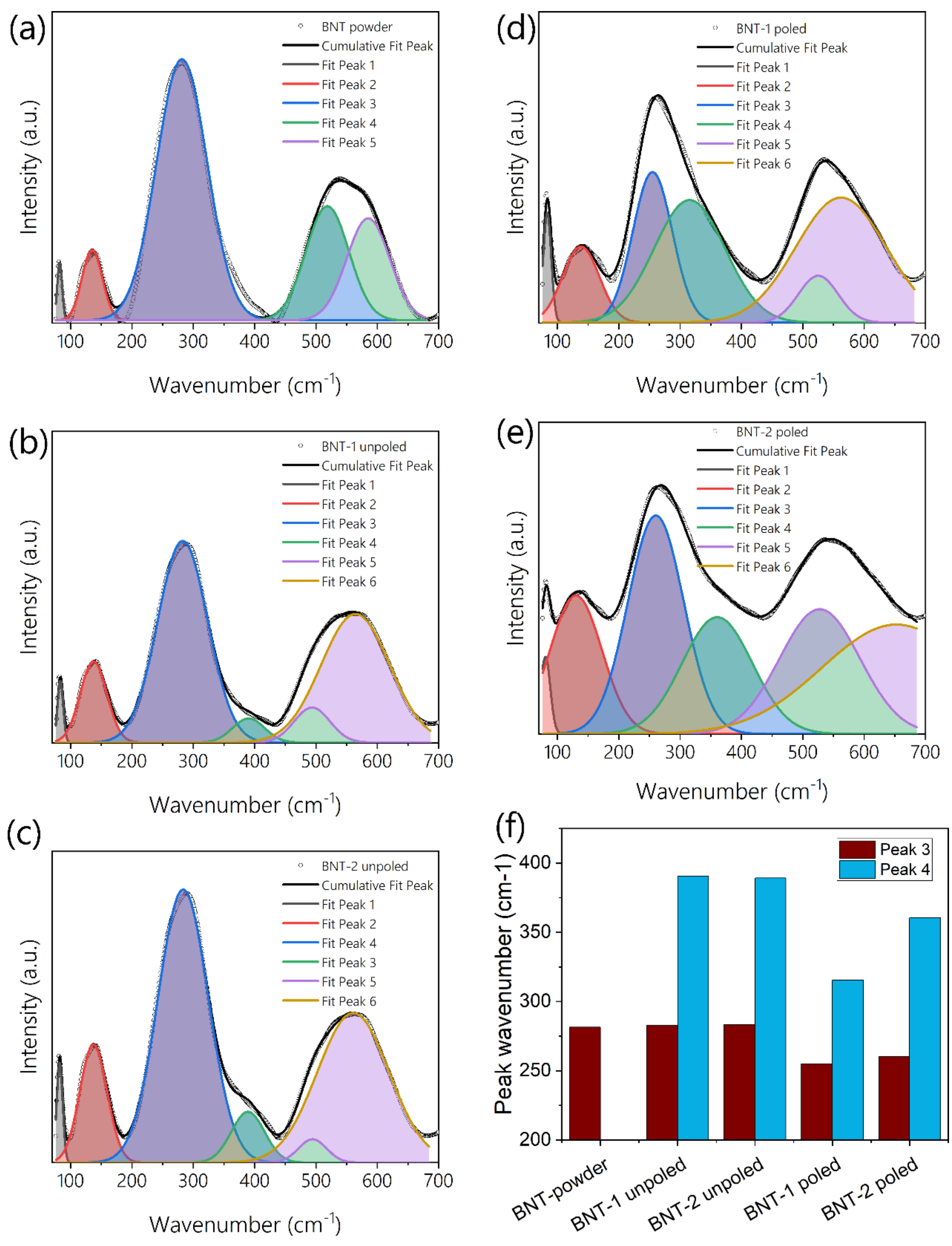

Supplementary Fig. 18. Raman spectra of BNT samples. (a) BNT-powder, (b) unpoled BNT-1, (c) unpoled BNT-2, (d) poled BNT-1, (e) poled BNT-2. Peaks are fitted for Ti-O, Bi-O, and Na-O vibration modes. (f) Comparison of Ti-O vibration mode for different BNT samples.

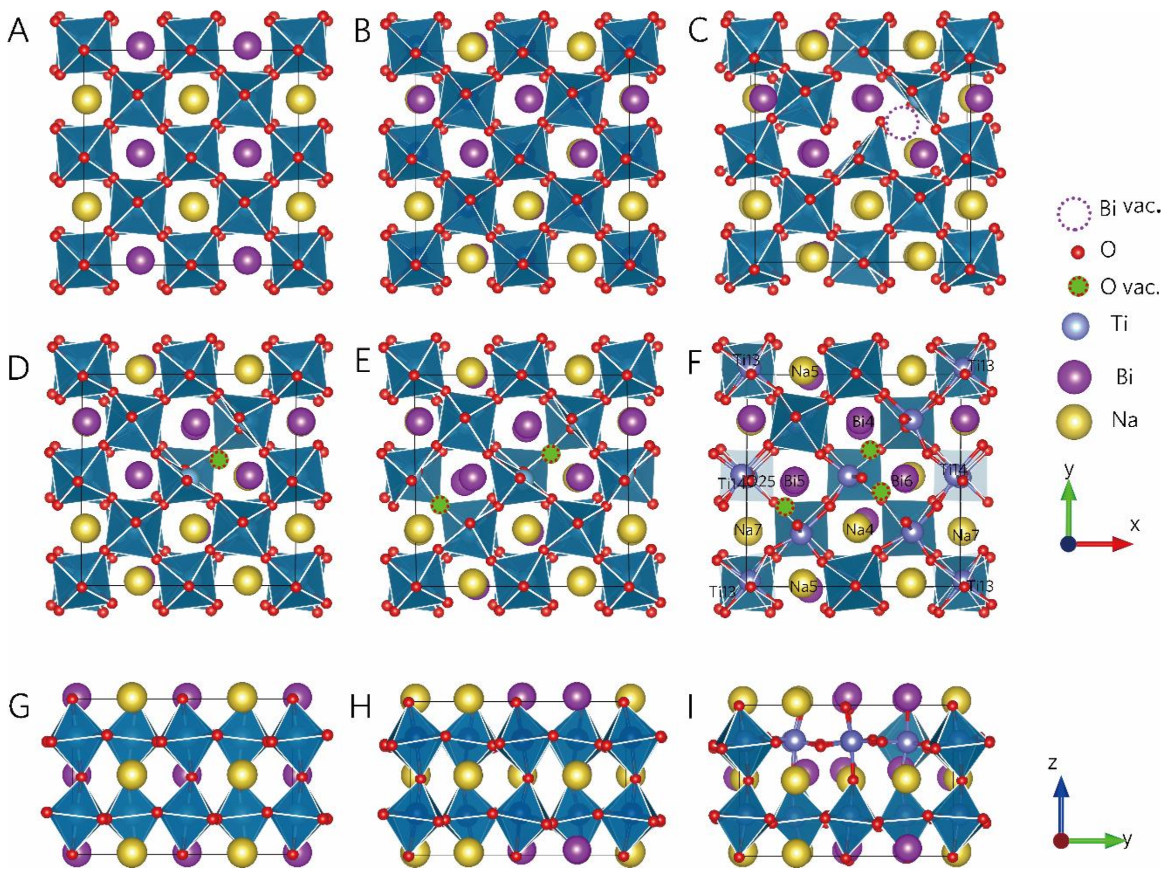

Supplementary Fig. 19. Relaxed geometry-lattice model of BNT. (A, G) Bi/Na order distribution, (B, H) Bi/Na disorder distribution, (C) BNT with 1/16 Bi vacancy, (D) BNT with 1/48 oxygen vacancy, (E) BNT with 2/48 oxygen vacancy, (F, I) BNT with 3/48 oxygen vacancy, (G) BNT with Bi/Na order distribution. (A-F) in the view of (001) plane, (G-I) in the view of (100) plane

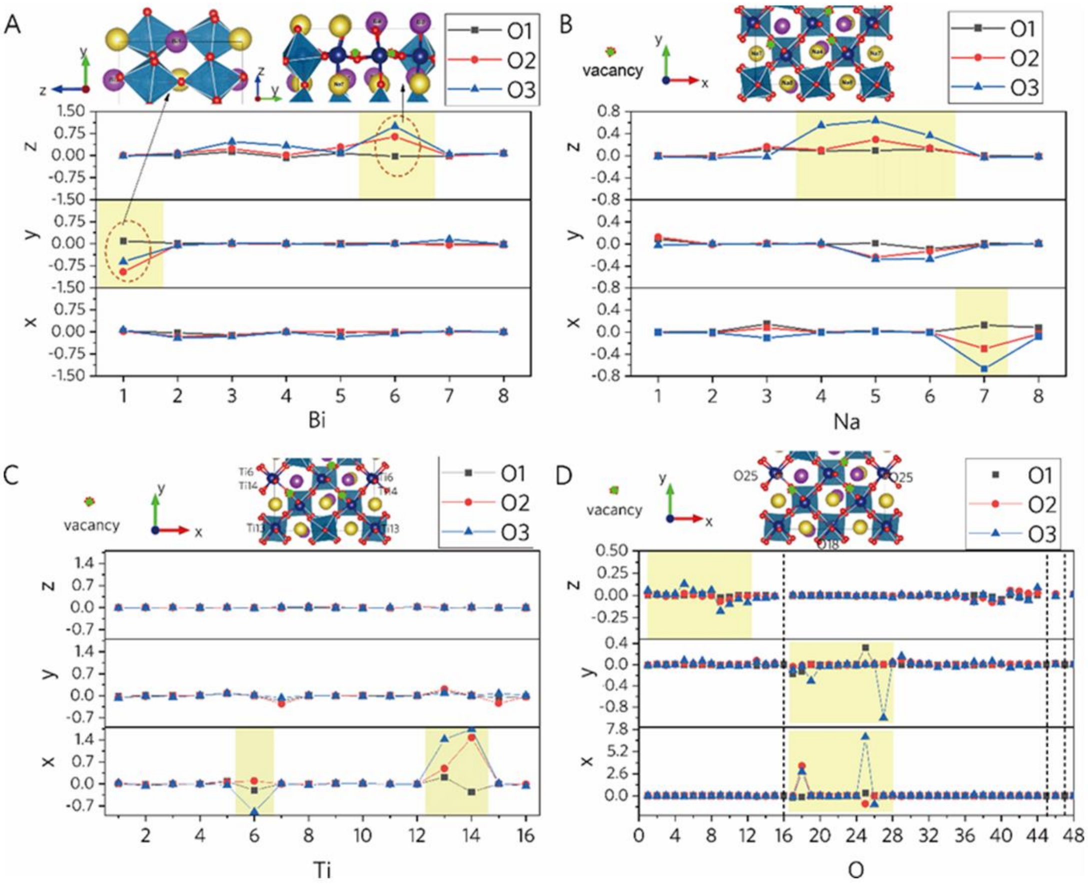

Supplementary Fig. 20. Relative ionic displacement of BNT with various amounts of oxygen vacancy in reference with that of BNT with zero vacancies in the fractional coordinate. (A) Bi ions, (B) Na ions, (C) Ti ions, (D) O ions. x, y, z represent the orientation of the coordinate axis in the crystal lattice. O1, O2, O3 represent 1/48, 2/48, and 3/48 oxygen vacancy. Yellow shades represent the large ratio corresponding to the inset geometry model.

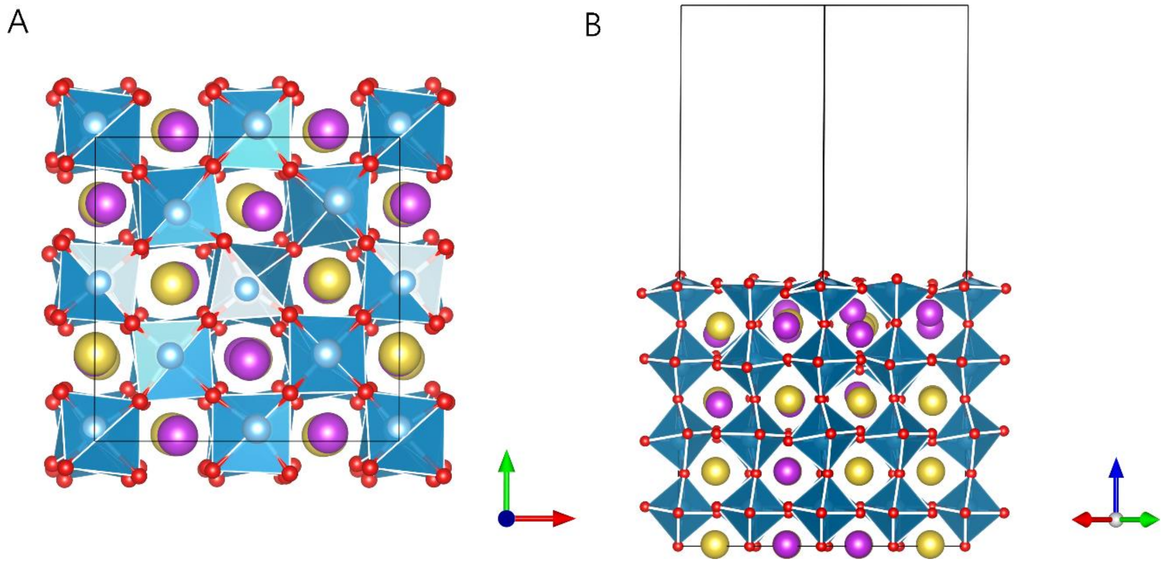

Supplementary Fig. 21. Relaxed geometry structure of BNT (001) surface with 1/48 oxygen vacancy in the view of (A) (001) plane and (B) (110) plane. The (001) surface is modeled based on the BNT (001) plane in Figure 5D.

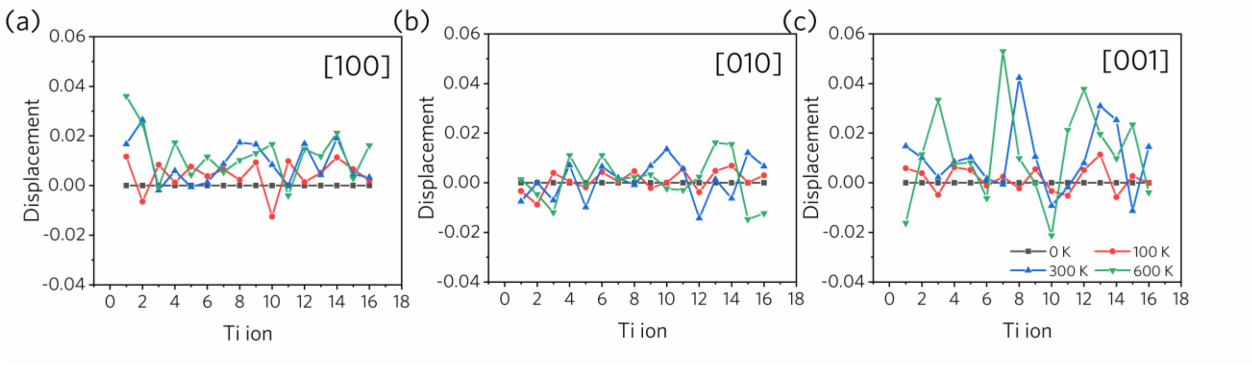

Supplementary Fig. 22. Temperature effects of ferroelectric ionic displacement of BNT. It is performed at 0, 100, 300 and 600K along [100], [010] and [001] direction.

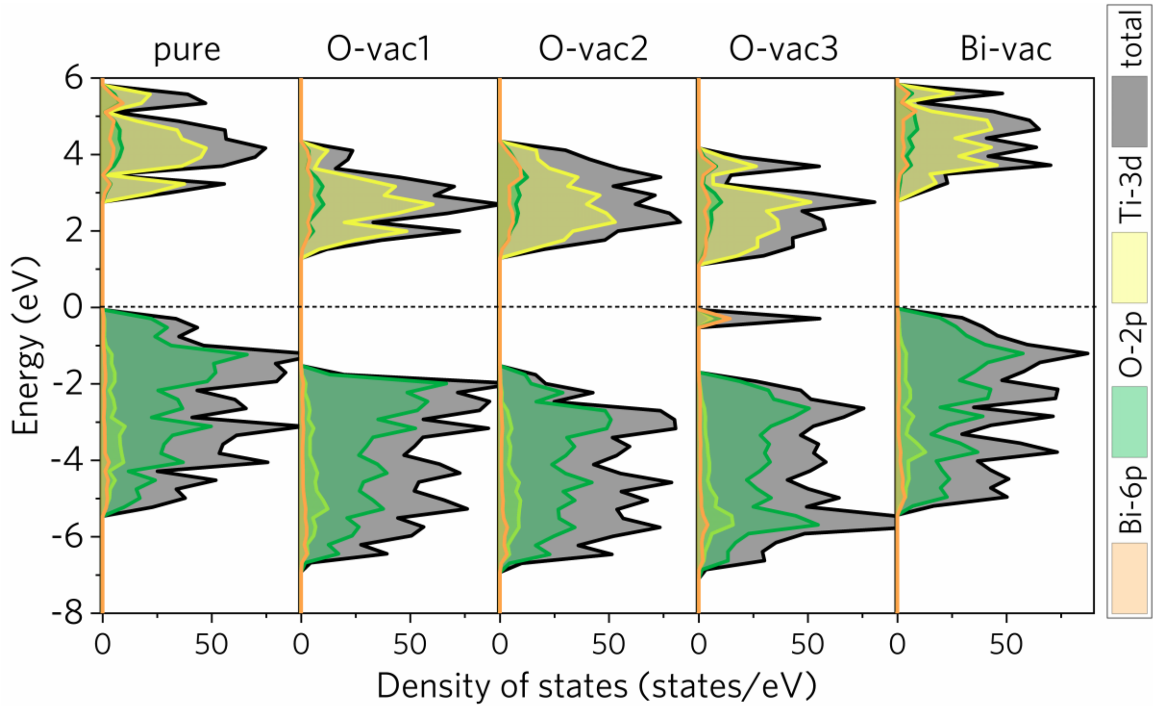

Supplementary Fig. 23. Total and partial density of states of BNT with (1/48, 2/48, 3/48) oxygen and 1/16 bismuth vacancies. Fermi level is at 0 eV.

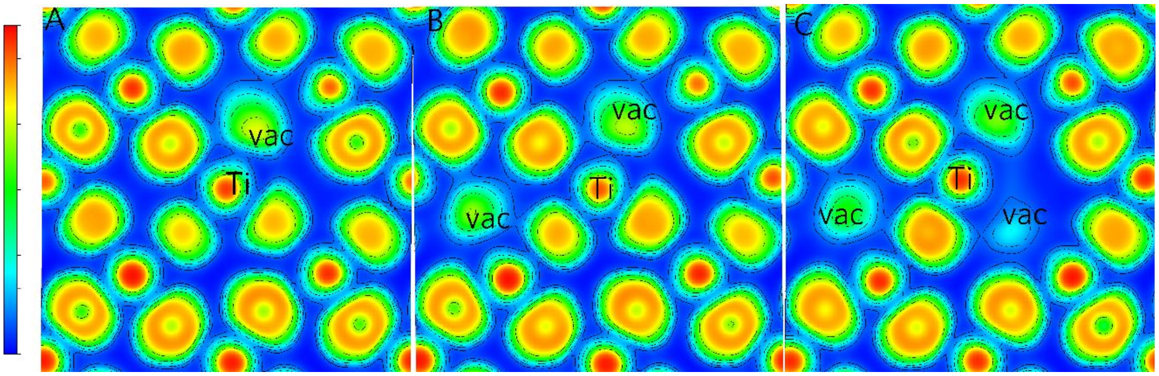

Supplementary Fig. 24. ELF of BNT. (A) BNT with 1/48 oxygen vacancy, (B) BNT with 2/48 oxygen vacancy, (C) BNT with 3/48 oxygen vacancy.

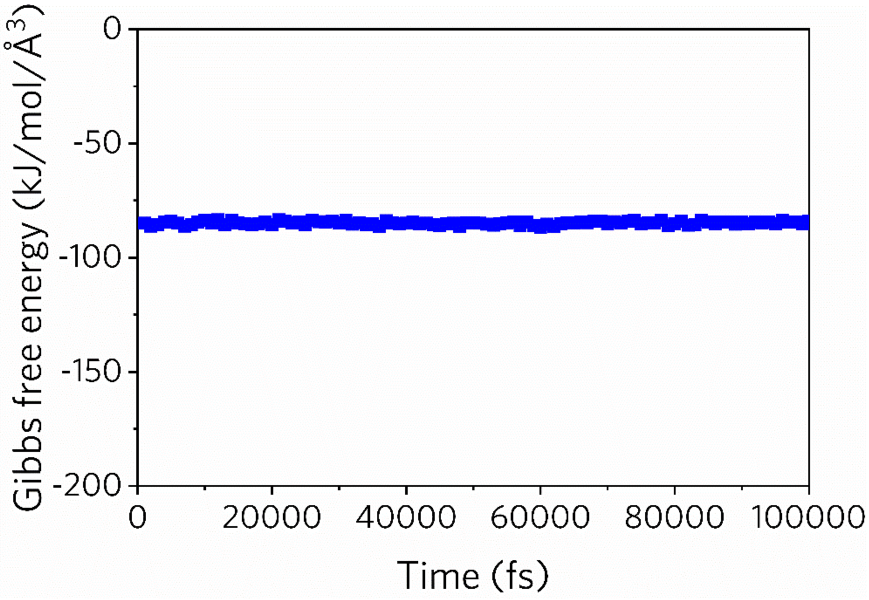

Supplementary Fig. 25. Gibbs free energy of BNT with disorder Bi/Na distribution at 1400K from MD simulation.

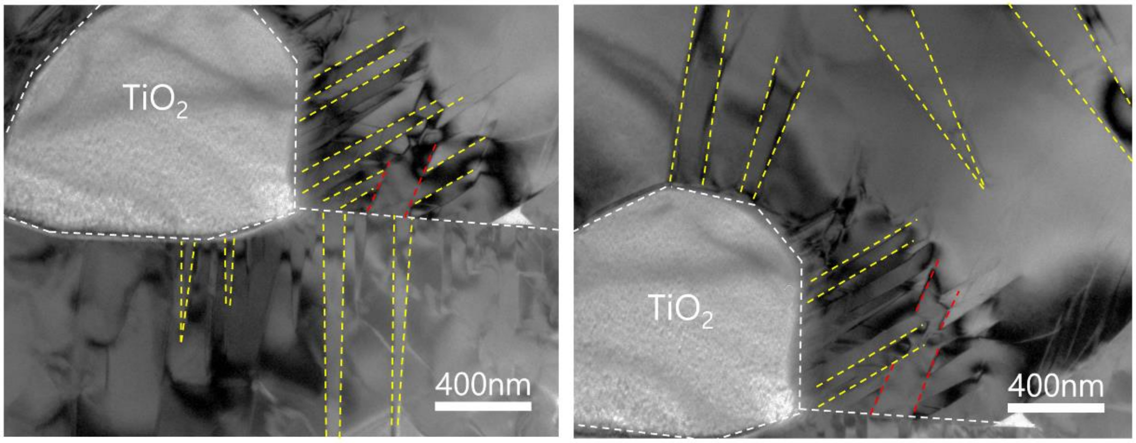

Supplementary Fig. 26. TEM images of the domains of BNT-TiO2 ceramics.

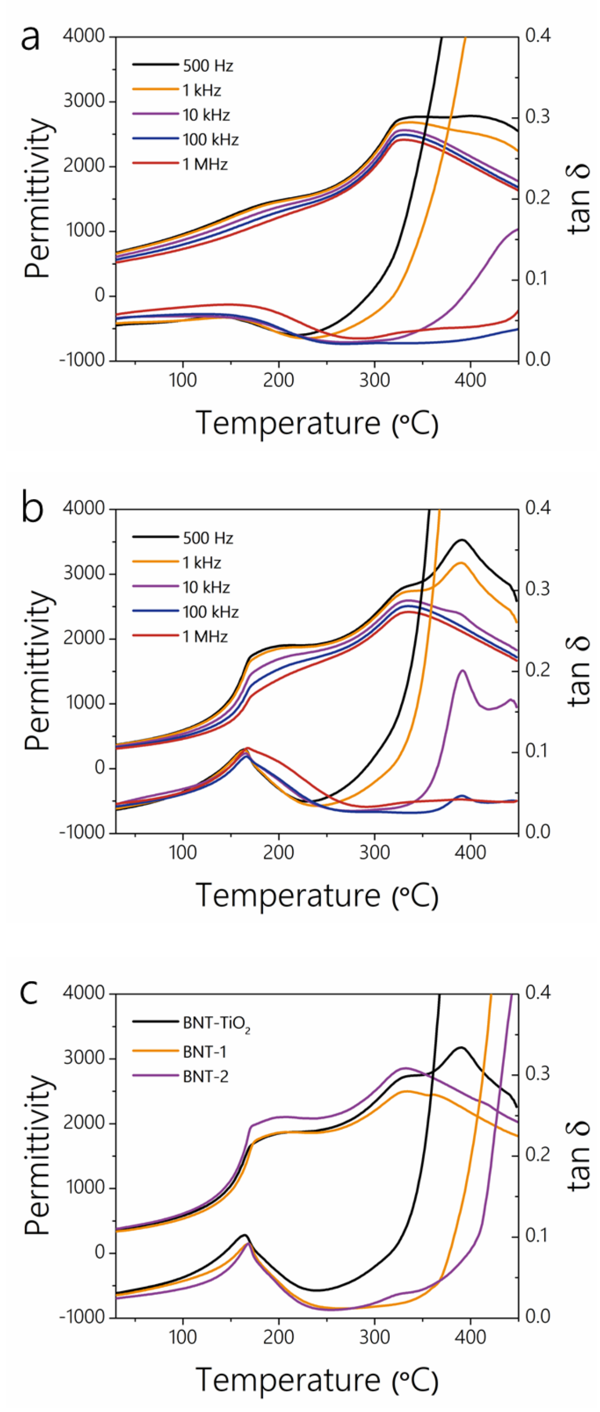

Supplementary Fig. 27. (A) Temperature dependence of dielectric constant (εr) and loss (tan δ). (A) BNT-TiO2, (B) polarized BNT-TiO2, and (C) a comparison of polarized BNT-1, BNT-2 and BNT-TiO2.

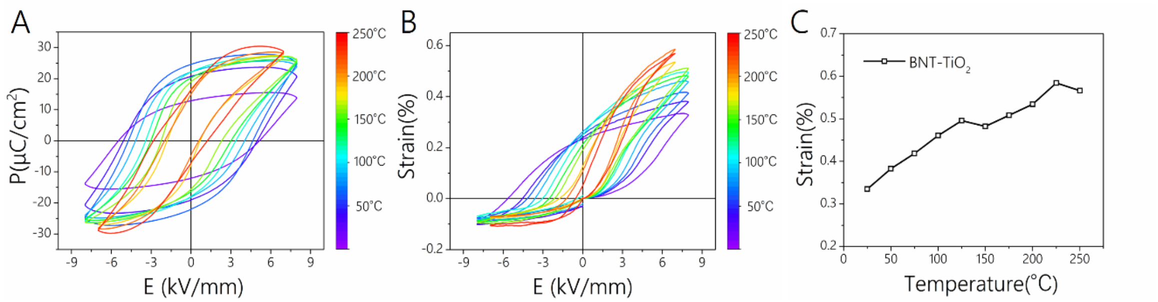

Supplementary Fig. 28. Piezoelectric performances of BNT-TiO_2 ceramics. (A) P-E hysteresis loops and (B) Bipolar strain curves of BNT-TiO_2 at different temperatures. (C) The maximum strain of BNT-TiO_2 at different temperatures.

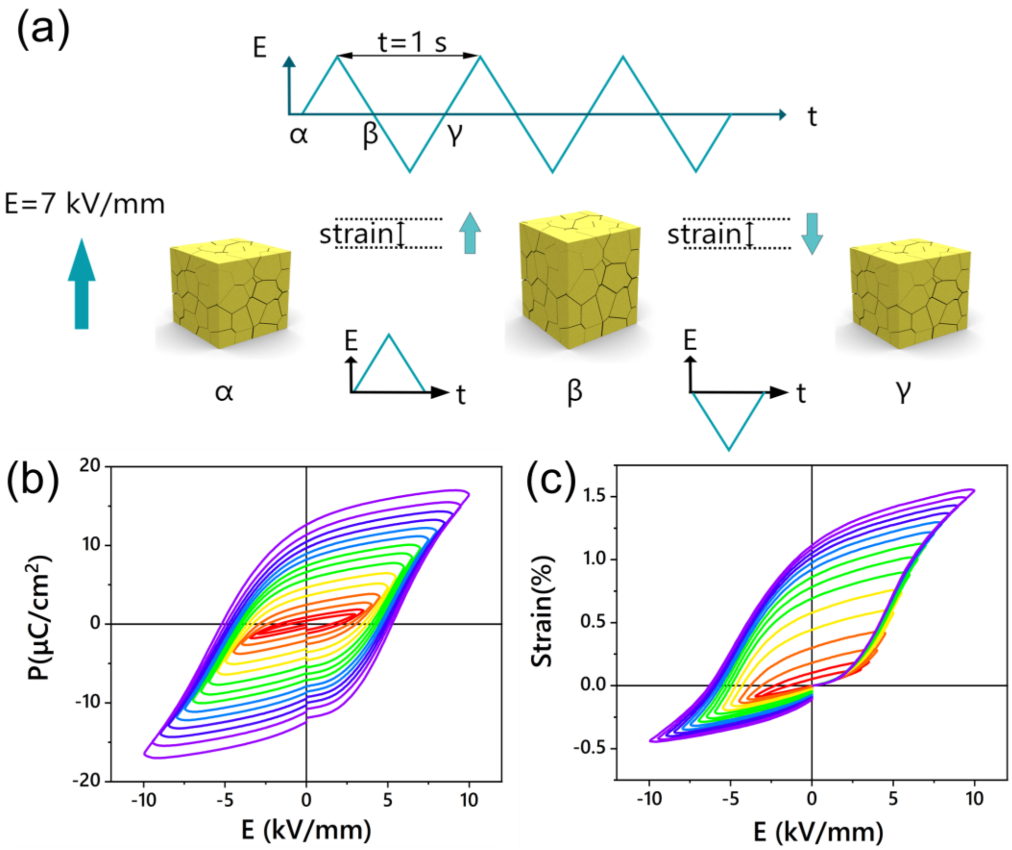

Supplementary Fig. 29. Strain memory effects of BNT-2 ceramics. (a) Schematic diagram of strain memory effect of BNT-2 ceramics. (b) Hysteresis curves at the different electric fields. (c) Bipolar strain curves at the different electric fields.

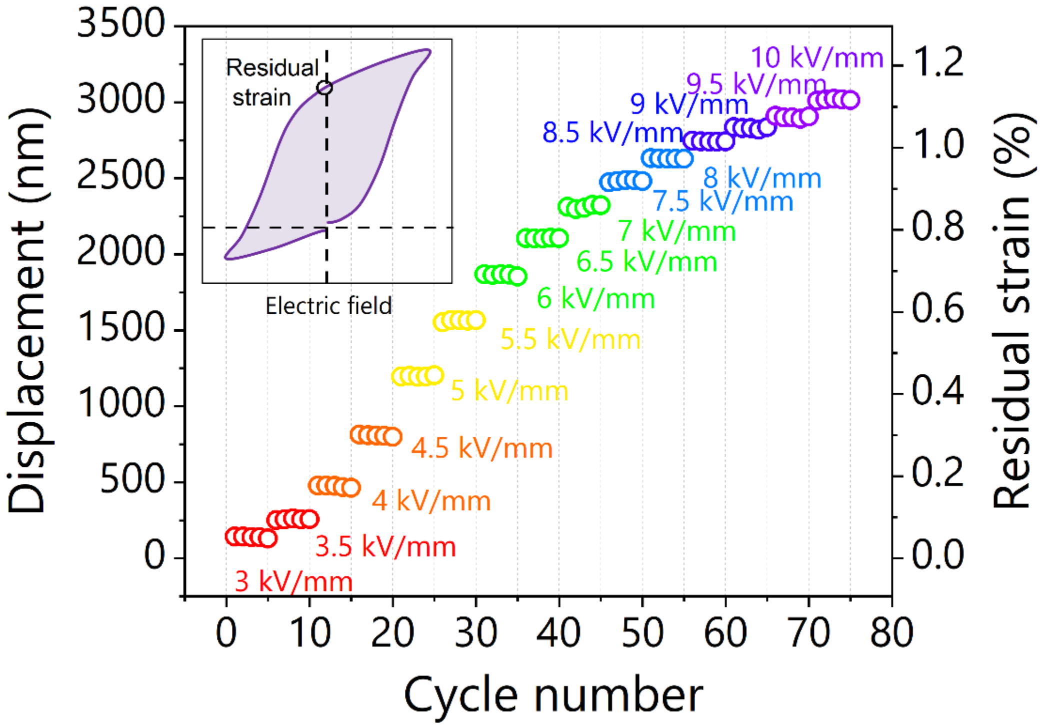

Supplementary Fig. 30. Strain memory effects of BNT-2 ceramics under different electric fields.

Supplementary Table 1. Element content testing by ICP-OES.

	BNT-1	BNT-2	unit
**Bi**	49.73	50.06	wt%
**Na**	5.203	5.193	wt%
**Ti**	22.86	23.12	wt%

Supplementary Table 2. Maximum electrostrain of state-of-the-art lead-based and lead-free polycrystalline piezoelectric. The present work shows the highest values in both room temperature and high-temperature regions. The electrostrain of present lead-free polycrystalline piezoelectric is higher than lead-based piezoelectric ceramics.

Materials	S_{max} (%)	Temp. (℃)	Structure	Lead-free	Ref.
BMT-PMN-PT	0.39	25	Textured ceramic	No	1
PYN-PMN-PT	0.18	25	Textured ceramic	No	2
BMT-PMN-PT	0.42	25	Textured ceramic	No	3
PSN-PMN-PT	0.13	25	Polycrystalline ceramic	No	4
PZN-8% PT	1.7	25	Single crystal	No	5
PMN-PT	0.28	25	Textured ceramic	No	6
PMN-PT	0.14	25	Polycrystalline ceramic	No	7
PMN-PT	0.23	25	Polycrystalline ceramic	No	8
PMN-PT	0.52	55	Single crystal	No	9
BFO-PT-La	1.3	25	Polycrystalline ceramic	No	10
BCZT	0.48	25	Polycrystalline ceramic	Yes	11
Fe-BT	0.75	25	Single crystal	Yes	12
KNN	0.9	25	Single crystal	Yes	13
BNKT-SBTZ6	0.72	25	Polycrystalline ceramic	Yes	14

BNT-Nb	0.7	25	Polycrystalline ceramic	Yes	15
BF-BHT	0.58	25	Polycrystalline ceramic	Yes	16
Sb-BNKT	0.46	25	Polycrystalline ceramic	Yes	17
NBKT-5FN/NKBT-4ST	0.25	25	Polycrystalline ceramic	Yes	18
BCZ-BNT	0.41	25	Polycrystalline ceramic	Yes	19
BNBT-YN	0.45	25	Polycrystalline ceramic	Yes	20
Ce-BNT-BT	0.4	25	Polycrystalline ceramic	Yes	21
NiMn-BNBT	0.46	25	Polycrystalline ceramic	Yes	22
KNBT	0.6	25	Polycrystalline ceramic	Yes	23
$Bi_4Ti_3O_{12}$	0.14	25	Textured ceramic	Yes	24
BNT-2	1.5	25	Polycrystalline ceramic	Yes	This work
BFO-BTO	0.37	180	Polycrystalline ceramic	Yes	25
BNF-BT	0.43	150	Polycrystalline ceramic	Yes	26
BCZT	0.18	80	Polycrystalline ceramic	Yes	11
KNN-BLT-BZ6	0.12	190	Polycrystalline ceramic	Yes	27
BiKFeO3-BT	0.26	105	Polycrystalline ceramic	Yes	28
LF4T	0.14	160	Textured ceramic	Yes	29
NBT-BT	0.35	175	Polycrystalline ceramic	Yes	30
BNT-Nb	0.59	100	Polycrystalline ceramic	Yes	15

BNT-2	2.3	220	polycrystalline ceramic	Yes	This work
PLZST	0.41	190	Polycrystalline ceramic	No	[31]
PZT4	0.16	160	Polycrystalline ceramic	No	[29]

Supplementary Table 3. Raman fitting parameters of BNT ceramics. xc is the center of peaks, w is the full width at half maxima(FWHM)

	**Bi-O**		**Na-O**		**Ti-O**				**[TiO6] octahedra**			
**Samples**	Peak 1		Peak 2		Peak 3		Peak 4		Peak 5		Peak 6	
	xc	w	xc	w	xc	w	xc	w	xc	w	xc	w
**BNT-powder**	82	9	135	39	281	97			519	83	585	83
**BNT-1 unpoled**	83	11	137	51	283	96	390	62	494	69	564	131
**BNT-2 unpoled**	82	12	137	52	284	97	389	64	495	54	561	138
**BNT-1 poled**	83	15	138	71	255	76	315	136	525	77	563	173
**BNT-2 poled**	81	19	130	100	260	107	361	135	528	154	653	286

Supplementary Table 4. Relative ionic displacement of Ti ions of BNT. Various amounts of oxygen vacancies in reference with that of pure BNT in the fractional coordinates are listed. x, y, z represent the orientation of the coordinate axis in the crystal lattice.

Atom	1/48 vacancy			2/48 vacancy			3/48 vacancy		
	x	y	z	x	y	z	x	y	z
**Ti1**	0%	0%	0%	3%	-4%	0%	3%	-7%	-1%
**Ti2**	-1%	0%	0%	-3%	-1%	0%	-6%	-1%	0%
**Ti3**	0%	0%	0%	0%	0%	0%	0%	-4%	1%
**Ti4**	1%	1%	0%	0%	2%	-1%	0%	1%	0%
**Ti5**	9%	7%	0%	5%	9%	0%	-4%	8%	1%
**Ti6**	-20%	0%	0%	10%	1%	-1%	-89%	2%	-2%
**Ti7**	1%	-17%	-1%	1%	-25%	0%	1%	-6%	3%
**Ti8**	0%	0%	-1%	0%	0%	3%	-4%	1%	2%
**Ti9**	1%	0%	-1%	2%	0%	1%	2%	0%	0%
**Ti10**	1%	0%	-1%	1%	0%	0%	2%	-1%	-2%
**Ti11**	0%	1%	-2%	0%	-1%	-1%	0%	-2%	1%
**Ti12**	-1%	0%	3%	-1%	0%	3%	0%	-1%	2%
**Ti13**	21%	15%	0%	50%	21%	0%	142%	9%	-1%
**Ti14**	-26%	0%	0%	147%	2%	1%	175%	1%	1%
**Ti15**	1%	-6%	0%	1%	-24%	0%	2%	8%	-1%
**Ti16**	-2%	-3%	-1%	-1%	-3%	-1%	-7%	1%	0%

Supplementary Table 5. Relative ionic displacement of Bi and Na ions of BNT. Various amounts of oxygen vacancies in reference with that of pure BNT in the fractional coordinates are listed. x, y, z represent the orientation of the coordinate axis in the crystal lattice.

Atom	1/48 vacancy			2/48 vacancy			3/48 vacancy		
	x	y	z	x	y	z	x	y	z
**Bi1**	2%	9%	0%	2%	-96%	0%	6%	-62%	-2%
**Bi2**	-3%	1%	-1%	-16%	-5%	8%	-21%	-6%	6%
**Bi3**	-11%	0%	13%	-12%	1%	23%	-17%	2%	47%
**Bi4**	0%	0%	-7%	0%	-1%	1%	-1%	1%	33%
**Bi5**	-2%	1%	7%	-1%	-1%	28%	-17%	-4%	9%
**Bi6**	0%	1%	-2%	-2%	0%	64%	-6%	-1%	99%
**Bi7**	1%	-2%	-2%	0%	-5%	-1%	3%	15%	4%
**Bi8**	-1%	-3%	8%	-1%	-4%	7%	0%	-3%	7%
**Na1**	0%	8%	0%	0%	12%	-1%	-1%	-2%	-1%
**Na2**	0%	0%	0%	-2%	-1%	-2%	-1%	0%	-4%
**Na3**	15%	1%	12%	8%	1%	17%	-11%	-1%	-2%
**Na4**	0%	-1%	9%	-1%	0%	10%	-1%	2%	55%
**Na5**	2%	1%	9%	1%	-24%	29%	1%	-28%	64%
**Na6**	0%	-9%	12%	0%	-13%	14%	-1%	-28%	36%
**Na7**	13%	1%	0%	-30%	-2%	-2%	-67%	-2%	-3%
**Na8**	8%	0%	-1%	-3%	1%	-2%	-8%	1%	-2%

Supplementary Table 6. Relative ionic displacement of O ions of BNT. Various amounts of oxygen vacancies in reference with that of pure BNT in the fractional coordinates are listed. x, y, z represent the orientation of the coordinate axis in the crystal lattice.

Atom	1/48 vacancy			2/48 vacancy			3/48 vacancy		
	x	y	z	x	y	z	x	y	z
**O1**	0%	-1%	0%	1%	-2%	2%	1%	-2%	6%
**O2**	1%	0%	1%	1%	0%	1%	2%	0%	1%
**O3**	0%	0%	0%	1%	-1%	-1%	0%	-1%	1%
**O4**	1%	0%	0%	1%	0%	0%	1%	0%	2%
**O5**	0%	0%	0%	0%	3%	2%	-4%	8%	13%
**O6**	0%	0%	1%	0%	1%	1%	-2%	2%	5%
**O7**	0%	1%	2%	0%	1%	-1%	0%	7%	2%
**O8**	0%	0%	1%	1%	0%	0%	0%	2%	6%
**O9**	-1%	0%	-2%	3%	-1%	-7%	6%	-3%	-18%
**O10**	0%	0%	-2%	1%	1%	-5%	3%	-1%	-10%
**O11**	0%	0%	0%	0%	1%	-1%	2%	-3%	-4%
**O12**	0%	0%	0%	0%	0%	-1%	1%	-1%	-8%
**O13**	-2%	2%	0%	-6%	7%	0%	-4%	5%	-3%
**O14**	-1%	0%	0%	-3%	0%	-2%	-3%	1%	-3%
**O15**	0%	1%	0%	0%	3%	-1%	1%	2%	-1%
**O16**									
**O17**	-17%	5%	0%	3%	-4%	0%	-10%	-10%	0%
**O18**	-12%	0%	0%	351%	-1%	0%	284%	-3%	0%
**O19**	0%	2%	0%	2%	0%	0%	1%	-30%	-1%
**O20**	-1%	-1%	0%	5%	-2%	1%	1%	-3%	1%
**O21**	-1%	0%	0%	0%	0%	0%	-7%	-3%	0%
**O22**	-2%	0%	0%	0%	0%	0%	-3%	0%	0%
**O23**	0%	0%	0%	1%	-1%	0%	-1%	1%	-1%
**O24**	0%	-1%	-1%	1%	-1%	-1%	0%	-2%	-1%
**O25**	32%	0%	0%	-93%	1%	0%	690%	1%	0%
**O26**	0%	0%	0%	0%	2%	-1%	-99%	1%	-1%
**O27**	0%	0%	0%	0%	1%	0%	2%	-100%	-1%
**O28**	1%	1%	0%	1%	4%	-1%	11%	4%	-2%
**O29**	0%	0%	0%	-2%	9%	1%	-1%	16%	1%
**O30**	0%	0%	0%	-1%	4%	0%	3%	3%	-1%
**O31**	0%	0%	0%	0%	2%	-1%	2%	1%	0%
**O32**	0%	0%	1%	0%	0%	1%	1%	1%	1%
**O33**	0%	0%	0%	0%	-2%	-1%	0%	-5%	-1%
**O34**	0%	0%	-1%	-1%	0%	-2%	-1%	0%	0%
**O35**	0%	-1%	0%	-1%	-1%	-1%	0%	-4%	2%
**O36**	0%	0%	0%	-1%	-1%	-2%	-1%	-1%	-2%
**O37**	-1%	0%	0%	3%	2%	-7%	14%	5%	-7%
**O38**	-1%	0%	0%	0%	1%	-3%	7%	1%	1%

**O39**	0%	0%	-2%	1%	1%	-8%	4%	7%	-4%
**O40**	1%	0%	-5%	1%	0%	-7%	3%	2%	-8%
**O41**	0%	1%	1%	0%	1%	6%	-2%	-6%	3%
**O42**	0%	0%	-2%	1%	0%	5%	1%	-1%	-2%
**O43**	0%	0%	-2%	0%	1%	2%	-1%	-4%	-5%
**O44**	0%	0%	1%	0%	0%	3%	0%	-2%	9%
**O45**									
**O46**	2%	0%	0%	3%	1%	2%	3%	1%	-1%
**O47**									
**O48**	1%	0%	1%	2%	0%	2%	2%	0%	1%

Supplementary Table 7. Relative ionic displacement of O ions of BNT (001) surface with 1/48 oxygen vacancy. It is performed under the electric field of 0.01 V/Å in reference with that under zero field in the fractional coordinates. x, y, z represent the orientation of the coordinate axis in the crystal lattice.

Atom label	x	y	z
**1**	0%	0%	1%
**2**	0%	4%	4%
**3**	0%	0%	0%
**4**	5%	-3%	5%
**5**	0%	0%	0%
**6**	0%	2%	-2%
**7**	-3%	4%	-2%
**8**	16%	-10%	-1%
**9**	0%	0%	0%
**10**	-152%	-92%	1%
**11**	0%	0%	0%
**12**	0%	-7%	1%
**13**	0%	0%	0%
**14**	3006%	267%	0%
**15**	0%	0%	0%
**16**	7%	15%	1%
**17**	-1%	-3%	-1%
**18**	6%	13%	1%
**19**	0%	0%	0%
**20**	5%	0%	0%
**21**	0%	0%	0%
**22**	2%	2%	2%
**23**	-3%	-1%	3%
**24**	-3%	-3%	3%
**25**	1%	0%	0%
**26**	-1%	2%	2%
**27**	0%	0%	0%
**28**	9%	-1%	0%
**29**	0%	0%	0%
**30**	-1%	1%	0%
**31**	1%	0%	0%
**32**	25%	-2%	1%
**33**	0%	0%	0%
**34**	382%	-2%	1%
**35**	0%	0%	0%
**36**	3%	0%	0%
**37**	0%	0%	0%

**38**	-1%	3%	1%
**39**	0%	0%	0%
**40**	3%	1%	0%
**41**	1%	0%	0%
**42**	8%	3%	1%
**43**	0%	0%	0%
**44**	-8%	-1%	-1%
**45**	0%	0%	0%
**46**	3%	1%	0%
**47**	2%	0%	0%
**48**	3%	-2%	1%
**49**	1%	-2%	2%
**50**	0%	2%	1%
**51**	0%	0%	0%
**52**	0%	2%	1%
**53**	0%	0%	0%
**54**	1%	1%	2%
**55**	0%	1%	1%
**56**	3%	-2%	1%
**57**	0%	0%	0%
**58**	3%	-13%	1%
**59**	0%	0%	0%
**60**	1%	0%	1%
**61**	0%	0%	0%
**62**	-1%	718%	1%
**63**	0%	0%	0%
**64**	0%	1%	1%
**65**	0%	-2%	0%
**66**	3%	8%	0%
**67**	0%	0%	0%
**68**	1%	0%	1%
**69**	0%	0%	0%
**70**	0%	1%	4%
**71**	1%	0%	1%
**72**	1%	0%	0%
**73**	0%	0%	2%
**74**	1%	2%	8%
**75**	0%	0%	0%
**76**	2%	0%	4%
**77**	0%	0%	0%
**78**	-2%	2%	-5%
**79**	1%	0%	0%
**80**	0%	0%	0%
**81**	-4%	-1%	1%
**82**	0%	0%	0%
**83**	1%	-4%	0%

**84**	0%	0%	0%
**85**	5%	1%	1%
**86**	0%	0%	0%
**87**	4%	10%	2%
**88**	0%	-1%	-1%
**89**	2%	2%	-1%
**90**	0%	0%	0%
**91**	0%	-1%	-1%
**92**	0%	0%	0%
**93**	2%	2%	5%
**94**	0%	0%	2%
**95**	-1%	-2%	4%

Supplementary Table 8. Relative ionic displacement of Bi and Na ions of BNT (001) surface with 1/48 oxygen vacancy. It is performed under the electric field of 0.01 V/Å in reference with that under zero field in the fractional coordinates. x, y, z represent the orientation of the coordinate axis in the crystal lattice.

**Na**	**x**	**y**	**z**	**Bi**	**x**	**y**	**z**
**1**	0%	0%	0%	1	1%	107%	3%
**2**	0%	0%	0%	2	1%	5%	-3%
**3**	0%	0%	0%	3	0%	0%	0%
**4**	2%	36%	1%	4	0%	0%	0%
**5**	0%	0%	0%	5	2%	0%	3%
**6**	-100%	1%	1%	6	0%	0%	0%
**7**	0%	0%	0%	7	58%	0%	0%
**8**	6%	1%	-1%	8	3%	25%	-2%
**9**	93%	1%	1%	9	1%	1%	2%
**10**	0%	0%	0%	10	0%	0%	0%
**11**	0%	0%	0%	11	0%	0%	0%
**12**	0%	-10%	1%	12	3%	-5%	2%
**13**	2%	4%	1%	13	0%	0%	0%
**14**	0%	0%	0%	14	0%	0%	0%
**15**	0%	0%	0%	15	-2%	1%	-2%
**16**	0%	0%	0%	16	4%	0%	1%

Supplementary Table 9. Relative ionic displacement of Ti ions of BNT (001) surface with 1/48 oxygen vacancy. It is performed under the electric field of 0.01 V/Å in reference with that under zero field in the fractional coordinates. x, y, z represent the orientation of the coordinate axis in the crystal lattice.

Ti	x	y	z
**1**	1%	0%	0%
**2**	5%	4%	1%
**3**	0%	0%	0%
**4**	50%	16%	0%
**5**	0%	0%	0%
**6**	2%	0%	1%
**7**	52%	2%	0%
**8**	-100%	-23%	2%
**9**	1%	0%	0%
**10**	5%	0%	1%
**11**	0%	0%	0%
**12**	11%	0%	0%
**13**	0%	0%	0%
**14**	3%	0%	0%
**15**	14%	1%	0%
**16**	-98%	0%	1%
**17**	0%	-2%	0%
**18**	1%	2%	1%
**19**	0%	0%	0%
**20**	0%	-14%	1%
**21**	0%	0%	0%
**22**	1%	3%	1%
**23**	0%	11%	0%
**24**	3%	-8%	1%
**25**	0%	0%	-1%
**26**	3%	4%	2%
**27**	0%	0%	0%
**28**	2%	0%	1%
**29**	0%	0%	0%
**30**	1%	0%	0%
**31**	-1%	0%	0%
**32**	1%	0%	1%

Supplementary References

1 Jia, H., Liang, Z., Li, Z., Li, F. & Wang, L. Texture technique to simultaneously achieve large electric field induced strain response and ultralow hysteresis in BMT-PMN-PT relaxor ferroelectric ceramics. *Scripta Materialia* **209**, 114409, doi:https://doi.org/10.1016/j.scriptamat.2021.114409 (2022).

2 Brova, M. J. *et al.* Relationship between composition and electromechanical properties of CuO-doped textured PYN-PMN-PT ceramics. *J. Eur. Ceram. Soc. (Netherlands)* **41**, 1230-1235, doi:https://doi.org/10.1016/j.jeurceramsoc.2020.09.048 (2021).

3 Jia, H., Zhu, W., Yang, S., Li, F. & Wang, L. Large electric field induced strain of $Bi(Mg_{1/2}Ti_{1/2})O_3$-$Pb(Mg_{1/3}Nb_{2/3})O_3$-$PbTiO_3$ ceramics textured by Template Grain Growth. *J. Eur. Ceram. Soc. (Netherlands)* **41**, 6406-6413, doi:https://doi.org/10.1016/j.jeurceramsoc.2021.06.058 (2021).

4 Dong, S. *et al.* Phase structures and electrical properties of Sm doped PSN-PMN-PT ceramics. *Journal of Alloys and Compounds* **881**, 160621, doi:https://doi.org/10.1016/j.jallcom.2021.160621 (2021).

5 Park, S.-E. & Shrout, T. R. Ultrahigh strain and piezoelectric behavior in relaxor based ferroelectric single crystals. *Journal of Applied Physics* **82**, 1804-1811, doi:10.1063/1.365983 (1997).

6 Yang, S. *et al.* Achieving both high electromechanical properties and temperature stability in textured PMN-PT ceramics. *Journal of the American Ceramic Society* **105**, 3322-3330, doi:https://doi.org/10.1111/jace.18293 (2022).

7 Wang, F., Wang, H., Yang, Q., Zhang, Z. & Yan, K. Fine-grained relaxor ferroelectric PMN-PT ceramics prepared using hot-press sintering method. *Ceramics International* **47**, 15005-15009, doi:https://doi.org/10.1016/j.ceramint.2021.02.055 (2021).

8 Yang, Y., Liu, C., Ji, Y., He, L. & Ren, X. Designed morphotropic relaxor boundary ceramic exhibiting large electrostrain and negligible hysteresis. *Acta Materialia* **208**, 116720, doi:https://doi.org/10.1016/j.actamat.2021.116720 (2021).

9 Li, J. *et al.* Influence of Phase Transitions on Electrostrictive and Piezoelectric Characteristics in PMN–30PT Single Crystals. *ACS Applied Materials & Interfaces* **13**, 38467-38476, doi:10.1021/acsami.1c07714 (2021).

10 Narayan, B. *et al.* Electrostrain in excess of 1% in polycrystalline piezoelectrics. *Nat Mater* **17**, 427-431, doi:10.1038/s41563-018-0060-2 (2018).

11 Hao, J., Bai, W., Li, W., Zhai, J. & Randall, C. Correlation Between the Microstructure and Electrical Properties in High-Performance $(Ba_{0.85}Ca_{0.15})(Zr_{0.1}Ti_{0.9})O_3$ Lead-Free Piezoelectric Ceramics. *Journal of the American Ceramic Society* **95**, 1998-2006, doi:10.1111/j.1551-2916.2012.05146.x (2012).

12 Ren, X. Large electric-field-induced strain in ferroelectric crystals by point-defect-mediated reversible domain switching. *Nat Mater* **3**, 91-94, doi:10.1038/nmat1051 (2004).

13 Hu, C. *et al.* Ultra-large electric field–induced strain in potassium sodium niobate crystals. *Science Advances* **6**, eaay5979, doi:10.1126/sciadv.aay5979 (2020).

14 Li, T. *et al.* Giant strain with low hysteresis in A-site-deficient $(Bi_{0.5}Na_{0.5})TiO_3$-based lead-free piezoceramics. *Acta Materialia* **128**, 337-344, doi:10.1016/j.actamat.2017.02.037 (2017).

15 Liu, X. & Tan, X. Giant Strains in Non-Textured $(Bi_{1/2}Na_{1/2})TiO_3$-Based Lead-Free Ceramics. *Adv Mater* **28**, 574-578, doi:10.1002/adma.201503768 (2016).

16 Zeng, F. *et al.* Effects of Hf4+ substitute on the enhanced electrostrain properties of 0.7BiFeO3-0.3BaTiO3-based lead-free piezoelectric ceramics. *Ceramics International* **48**, 10539-10546, doi:https://doi.org/10.1016/j.ceramint.2021.12.265 (2022).

17 Wang, X., Liu, X., Xue, H., Yin, J. & Wu, J. Temperature-independent large strain with small hysteresis in Sb-modified BNT-based lead-free ceramics. *Journal of the American Ceramic Society* **105**, 2116-2127, doi:https://doi.org/10.1111/jace.18227 (2022).

18 Fan, P. *et al.* Large strain under low driving field in lead-free relaxor/ferroelectric composite ceramics. *Journal of the American Ceramic Society* **102**, 4113-4126, doi:https://doi.org/10.1111/jace.16256 (2019).

19 Liu, X., Han, J., Huang, Y., Yin, J. & Wu, J. Large electrostrictive coefficient with high electro-strain dominated by modified ergodic state in BNT-based solid solutions. *Journal of the American Ceramic Society* **104**, 1391-1401, doi:https://doi.org/10.1111/jace.17537 (2021).

20 Wang, H. *et al.* Large electro-strain with excellent fatigue resistance of lead-free (Bi0.5Na0.5)0.94Ba0.06Ti1-x(Y0.5Nb0.5)xO3 perovskite ceramics. *Ceramics International* **47**, 17092-17098, doi:https://doi.org/10.1016/j.ceramint.2021.03.018 (2021).

21 Liu, X., Yin, J. & Wu, J. A new class of ion substitution to achieve high electrostrain under low electric field in BNT-based ceramics. *Journal of the American Ceramic Society* **104**, 6277-6289, doi:https://doi.org/10.1111/jace.18008 (2021).

22 Kong, Y., Li, X., Li, Z. & Hao, J. Temperature independent fatigue-free behavior in sodium bismuth titanate-based lead-free ceramics. *Scripta Materialia* **194**, 113678, doi:https://doi.org/10.1016/j.scriptamat.2020.113678 (2021).

23 Das Adhikary, G., Dwij, V., Senyshyn, A., Sathe, V. & Ranjan, R. Large nonlinear electrostrain and piezoelectric response in nonergodic $Na_{0.5}Bi_{0.5}TiO_3$: Synergy of structural disorder and tetragonal phase in proximity to a morphotropic phase boundary. *Physical Review Materials* **5**, 064414, doi:10.1103/PhysRevMaterials.5.064414 (2021).

24 He, X. *et al.* Textured $Bi_4Ti_3O_{12}$ Ceramics: One-Step Spark Plasma Sintering and Their Single-Crystal-Like Polar Anisotropy. *Advanced Engineering Materials* **n/a**, 2200058, doi:https://doi.org/10.1002/adem.202200058 (2022).

25 Zheng, T. & Wu, J. Perovskite $BiFeO_3$-$BaTiO_3$ Ferroelectrics: Engineering Properties by Domain Evolution and Thermal Depolarization Modification. *Advanced Electronic Materials*, doi:10.1002/aelm.202000079 (2020).

26 Wang, D. *et al.* Temperature dependent, large electromechanical strain in Nd-doped $BiFeO_3$ - $BaTiO_3$ lead-free ceramics. *Journal of the European Ceramic Society* **37**, 1857-1860, doi:10.1016/j.jeurceramsoc.2016.10.027 (2017).

27 Zhang, M.-H. *et al.* Thermally stable piezoelectric properties of (K, Na)NbO_3-based lead-free perovskite with rhombohedral-tetragonal coexisting phase. *Acta Materialia* **122**, 344-351, doi:10.1016/j.actamat.2016.10.011 (2017).

28 Kang, F. *et al.* High temperature 0.63BiFeO3-0.37BaTiO3 based ceramics with large electromechanical properties by simple A-site K+ doping. *Journal of Alloys and Compounds* **899**, 163289, doi:https://doi.org/10.1016/j.jallcom.2021.163289 (2022).

29 Yasuyoshi Saito *et al.* Lead-free piezoceramics. *Nature* **432**, 81-84, doi:10.1038/nature03008 (2004).

30 Yin, J., Zhao, C., Zhang, Y. & Wu, J. Ultrahigh strain in site engineering-independent $Bi_{0.5}Na_{0.5}TiO_3$-based relaxor-ferroelectrics. *Acta Materialia* **147**, 70-77, doi:10.1016/j.actamat.2018.01.054 (2018).

31 Ji, Y. *et al.* Reversible and High-Temperature-Stabilized Strain in (Pb,La)(Zr,Sn,Ti)O_3 Antiferroelectric Ceramics. *ACS Appl Mater Interfaces* **11**, 32135-32143, doi:10.1021/acsami.9b09552 (2019).
